# Functionalized Substrates for Reduced Nonradiative Recombination in Metal-Halide Perovskites

**DOI:** 10.1021/acs.jpclett.4c03307

**Published:** 2024-12-30

**Authors:** Guus J. W. Aalbers, Willemijn H. M. Remmerswaal, Ralph H. C. van den Heuvel, Laura Bellini, Lana M. Kessels, Christ H. L. Weijtens, Nick R. M. Schipper, Martijn M. Wienk, René A. J. Janssen

**Affiliations:** † Molecular Materials and Nanosystems & Institute for Complex Molecular Systems, Eindhoven University of Technology, P.O. Box 513, 5600 MB Eindhoven, The Netherlands; ‡ Dutch Institute for Fundamental Energy Research, De Zaale 20, 5612 AJ Eindhoven, The Netherlands

## Abstract

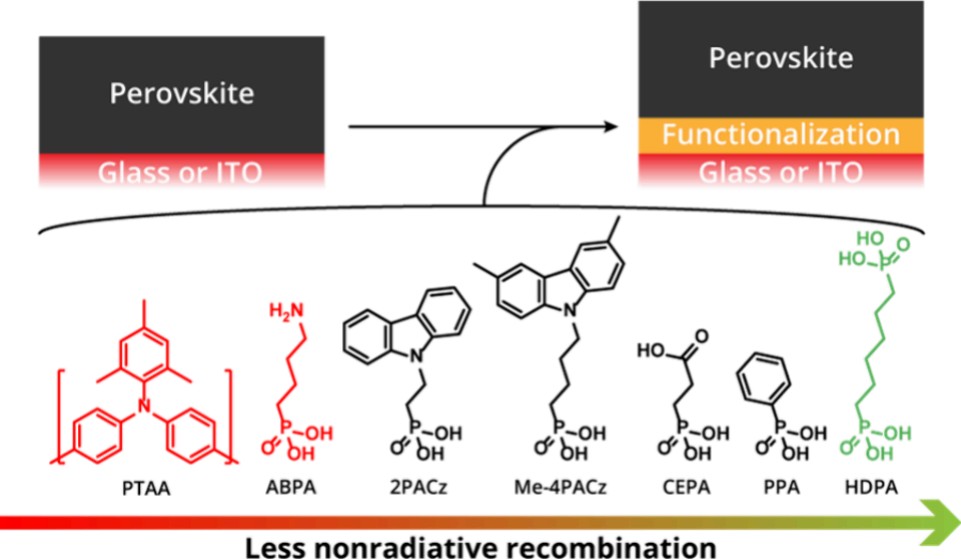

Reducing nonradiative recombination is crucial for minimizing voltage losses in metal-halide perovskite solar cells and achieving high power conversion efficiencies. Photoluminescence spectroscopy on complete or partial perovskite solar cell stacks is often used to quantify and disentangle bulk and interface contributions to nonradiative losses. Accurately determining the intrinsic loss in a perovskite layer is key to analyzing the origins of nonradiative recombination and developing defect engineering strategies. Here, we study perovskite films on glass and indium–tin-oxide-covered glass substrates, functionalized with a range of different molecules, using absolute and transient photoluminescence. We find that grafting these substrates with 1,6-hexylenediphosphonic acid (HDPA) effectively reduces the nonradiative losses in perovskite films for a series of perovskite semiconductors with bandgaps ranging from 1.26 to 2.28 eV. The results suggest that perovskites processed on HDPA-functionalized substrates suffer the least from nonradiative recombination and thus approach the properties of a defect-free semiconductor.

Metal-halide perovskite solar cells (PSCs) show great potential for single- and multiple-junction solar cell applications because of their excellent optoelectronic properties and easy bandgap tuning. Achieving a high open-circuit voltage (*V*_OC_) is essential for high efficiency. In PSCs, the *V*_OC_ values are mostly limited by electronic defect states in the perovskite bulk or at interfaces with charge transport layers and are often mediated via interstitials and vacancies.^1−3^ Understanding the location and origin of voltage losses in PSCs is essential to enhance *V*_OC_ and consequently their efficiency by defect engineering.

Voltage losses in PSCs originate from the radiative and nonradiative recombination of charge carriers. While radiative losses are unavoidable, nonradiative recombination is induced by defects (traps) and can be reduced by creating defect-free semiconductors and interfaces.^4^ Without nonradiative recombination, the solar cell reaches the radiative voltage limit (*V*_OC_^rad^), also known as the detailed-balance limit. Common techniques to assess nonradiative losses in semiconductors are steady-state and transient photoluminescence (PL) spectroscopy, which probe the radiative recombination of charge carriers. The internal voltage of a semiconductor under illumination equals the quasi-Fermi level splitting (QFLS or Δ*E*_F_), i.e., the difference between the Fermi levels of holes and electrons under nonequilibrium conditions.^5^ The nonradiative voltage loss is then given by the difference between the *V*_OC_^rad^ and Δ*E*_F_. For perovskites, the QFLS is often used to disentangle nonradiative voltage losses of a device stack into losses originating from the neat perovskite film or losses induced by the interface between the perovskite and charge transport layers (CTLs).^5−7^ By measuring the QFLS of partial PSC stacks, i.e., a neat perovskite film or the perovskite film adjacent to one or more CTLs, one can identify whether the dominating nonradiative recombination occurs in the perovskite bulk or at an interface and design and test defect-engineering strategies based on that insight.

Establishing the correct QFLS for the neat perovskite film is essential for correct interface loss analysis. For this purpose, neat perovskites are typically deposited as thin films on glass or quartz substrates,^8−11^ and any interface recombination is then assessed by inserting CTLs below or atop the perovskite film. However, because perovskite films cannot exist without interfaces, the “neat” perovskite film is also influenced by the substrate/perovskite and perovskite/air interfaces. This can result in an incorrect QFLS for the neat perovskite film and lead to inaccurate indications of the nonradiative interface losses caused by the CTLs.^12−15^ A good example of this problem was reported by Yuan et al.,^13^ who showed that a perovskite processed on a self-assembled monolayer (SAM) hole-transport layer (HTL) showed less nonradiative losses than the same “neat” perovskite film on a glass substrate. This inhibits any meaningful attempt to assess the nonradiative losses caused by the HTL, but the result highlights that depositing the perovskite film on the SAM reduces the nonradiative recombination in the bulk or at its interface with the substrate compared to that on glass. It has frequently been demonstrated that perovskite films deposited on different substrates or surfaces produce widely ranging nonradiative losses, e.g., for perovskite layers on various CTLs^5,9,15,16^ or quartz.^11^ The surface properties of the (functionalized) substrate are important because they can affect interfacial nonradiative recombination and the inherent quality of the perovskite layer deposited atop. This issue is scarcely addressed in the literature in studies where the nonradiative losses of partial PCS stacks are compared. The substrate that yields the highest possible QFLS for a neat perovskite film will be the best proxy for a defect-free semiconductor and is an essential starting point to investigate the effect of any subsequent bulk or interface passivation strategy.

This motivated us to study the effect of the substrate surface on the nonradiative recombination losses of perovskite films deposited on top. By functionalizing glass substrates with a range of different molecules, we show that the substrate, with and without functionalization, can induce significant nonradiative losses in the perovskite film. We find that grafting substrates with 1,6-hexylenediphosphonic acid (HDPA) vastly enhances the QFLS of the perovskite film on neat glass and glass covered with indium tin oxide (ITO). The general applicability of HDPA as a surface modifier to approach defect-free perovskite semiconductors is demonstrated by applying this surface functionalization to a range of different perovskite compositions with bandgaps between 1.26 and 2.28 eV. All perovskites tested showed the highest QFLS and longest PL decay time when deposited on HDPA-treated substrates. This sets HDPA surface functionalization as a new standard for voltage loss analyses of perovskite films and highlights that both glass and ITO-covered glass are not suitable as reference substrates when PL techniques are used.

We started our investigations by depositing triple-cation mixed-halide Cs_0.05_(FA_0.83_MA_0.17_)_0.95_Pb(I_0.83_Br_0.17_)_3_ (with MA = methylammonium and FA = formamidinium) perovskite films on neat or functionalized glass and ITO-covered glass. This perovskite has a bandgap of 1.63 eV and will be referred to as PVK-1.63 (Figure S1b). The molecules and materials used for substrate functionalization were (2-(9*H*-carbazol-9-yl)ethyl)phosphonic acid (2PACz), 4-aminobutylphosphonic acid (ABPA), phenylphosphonic acid (PPA), 2-carboxyethylphosphonic acid (CEPA), 1,6-hexylenediphosphonic acid (HDPA), (4-(3,6-dimethyl-9*H*-carbazol-9-yl)butyl)phosphonic acid (Me-4PACz), and poly[bis(4-phenyl)(2,4,6-trimethylphenyl)amine] (PTAA) (Figure 1). Among these, we differentiate between materials that exhibit selectivity for hole extraction (2PACz, Me-4PACz, and PTAA) and materials without charge selectivity (CEPA, PPA, ABPA, and HDPA). The first set has ionization potentials close to that of PVK-1.63 (5.5 eV), while these are much higher for the second category (>7.0 eV) as determined by ultraviolet photoelectron spectroscopy (UPS) (Figure S2a). Surface functionalization of glass and glass/ITO substrates alters the surface free energy (SFE) and results in the lowest SFE for Me-4PACz (∼39 mJ m^–2^) and the highest SFE for CEPA (∼72 mJ m^–2^) (Figure S3). The SFE plays a role in the wettability of the surface by the perovskite precursor solution during spin coating. The formation of the perovskite layer film may further be affected by specific interactions of the perovskite and its precursors with functional groups at the substrate surface (e.g., carboxylic acid, phosphonic acid, and amine) of which the conjugate ions can replace halide or A-site cations at the perovskite surface that interfaces with the SAM. In accordance with previous results,^17^ we find that substrates with low SFE result in incomplete coverage of the substrate and somewhat thinner (∼500 vs 525 nm) perovskite films compared to substrates with higher SFE, while the surface roughness shows small random variations between 11.0 and 14.5 nm (Figures S4 and S5). X-ray diffractometry (XRD) of PVK-1.63 films on functionalized glass or glass/ITO substrates revealed minor differences and no additional or disappearing reflections (Figure S6). Depending on the substrate, there is some variation in the relative intensity of the 2θ = 12.7° reflection of PbI_2_, but this is expected to have a minimal effect on the nonradiative loss.^18^ Moreover, as shown in Figure S7, there is no clear correlation between the ratio of the (100) perovskite and PbI_2_ Bragg peaks and the QFLS of such layers derived from the PL intensity.

**Figure 1:**
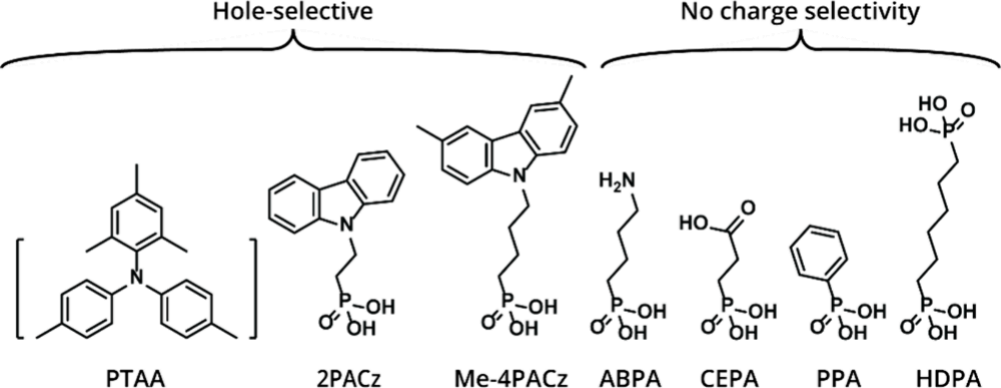
Structures of molecules and materials used to functionalize glass and glass/ITO substrates. Subdivided into hole-selective and materials without charge selectivity.

To evaluate the quality of the perovskite film and the nonradiative losses on each functionalized substrate, absolute steady-state PL (ss-PL) spectra were quantified by measuring the photon flux with a spectrally- and intensity-calibrated spectrometer. Measuring the absolute intensity of the PL allows for determining the QFLS of the deposited PVK-1.63 layers (Figure 2a), as explained in the Supporting Information. For PVK-1.63 deposited on neat glass or glass/ITO, the average QFLS values are 1135 ± 15 and 1114 ± 6 meV, respectively. This indicates significant nonradiative losses of ∼220 meV compared to the radiative voltage limit for this perovskite (*qV*_OC_^rad^ = 1340 meV).^12^ On functionalized glass substrates, the QFLS of PVK-1.63 increases compared to that on the neat glass substrates (Figure 2a), regardless of any charge selectivity of the surface functionalization. This indicates that modifying the surface by the materials shown in Figure 1 reduces the nonradiative losses by an improved substrate–perovskite interface or by an improved perovskite film formation, yielding a better, i.e., less-defective, semiconductor. The functionalization of glass/ITO substrates generally resulted in a higher QFLS for PVK-1.63 compared to functionalized glass, suggesting different wetting or crystallization processes occurring for the perovskite on the glass/ITO substrates.^19,20^ The coverage or anchoring of the SAM molecules on glass and glass/ITO can also differ, but generally, the SFE does not vary significantly between the two (Figure S3). This suggests that there is no significant difference in packing densities on glass and glass/ITO substrates for all seven surface treatments tested. Note that for ITO surface coverage to be effective in a perovskite device, the functionalization must also provide selective charge transport properties, in addition to reducing nonradiative losses.

**Figure 2:**
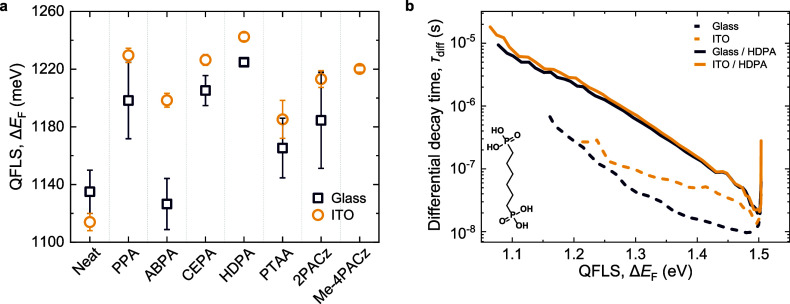
(a) QFLS of PVK-1.63 perovskite films deposited on functionalized glass and glass/ITO substrates determined from absolute ss-PL. (b) The differential decay time of PVK-1.63 perovskite film on neat or HDPA-functionalized glass or glass/ITO.

The fact that substrates functionalized with molecules with and without charge selectivity both improve the QFLS of PVK-1.63 indicates that the energetic properties of the functionalized substrate are less relevant for the increase in QFLS. In accordance, Figure S2b shows that there is no correlation between the QFLS and the ionization energies of the functionalized surfaces determined by UPS. Also, the SFE of the different functionalized glass and glass/ITO substrates show no correlation with the perovskite QFLS (Figure S8), ruling out the direct influence of substrate SFE on the QFLS of the perovskite semiconductor.

Out of the several functionalized surfaces tested, HDPA grafting resulted in the highest and most reproducible QFLS for PVK-1.63. In particular, the highest gain and reproducibility in QFLS (1225 ± 2 meV) was achieved when HDPA was grafted onto glass, improving the QFLS by 90 meV compared to neat glass. On an HDPA-covered glass/ITO substrate, PVK-1.63 showed the highest QFLS and reached 1242 ± 3 meV, which is only ∼100 meV below, and >92% of, the detailed-balance radiative voltage limit.

To gain a better understanding of the charge carrier recombination behavior leading to reduced nonradiative losses in PVK-1.63 films on HDPA-grafted substrates, transient PL (tr-PL) was used to study the temporal PL decay of PVK-1.63 films. The tr-PL decay is converted into a carrier-density-dependent decay time (τ_diff_) and is defined for high-level injection as^21^
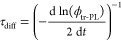
1where ϕ_tr-PL_ is the PL intensity and *t* is time. The differential decay time τ_diff_ can then be plotted as a function of the time-dependent QFLS. The QFLS relates to the carrier density and decreases with time after the excitation pulse via Δ*E*_F_(*t*) ∝ ln[ϕ_tr-PL_(*t*)] and is explained in the Supporting Information. Figure 2b shows τ_diff_ vs QFLS (Δ*E*_F_) for PVK-1.63 films on neat and HDPA-functionalized glass and glass/ITO substrates. It is evident that over the entire QFLS range, the differential decay time is considerably longer for the HDPA-functionalized substrates than for the neat substrates. At high QFLS, fast radiative recombination dominates for PVK-1.63 on the HDPA-covered substrates, resulting in a fast change of τ_diff_.^13^ On these substrates, the differential decay time is changing continuously with an almost constant slope following a power-law type behavior (τ_diff_ ∝ *t*^–α^), consistent with the behavior expected for an intrinsic semiconductor limited by shallow defects.^13^ On glass/ITO/HDPA substrates, PVK-1.63 gave longer detectable decay times reaching ∼20 μs, albeit with a small difference compared to glass/HDPA. HDPA-functionalization significantly enhances the detectable τ_diff_ compared with neat glass (∼700 ns). The fact that for PVK-1.63 on neat glass or glass/ITO the differential decay time is significantly shorter for every QFLS and does not follow a power-law relationship with time indicates that deep defects are limiting τ_diff_.^13^ These deep defects are absent when the HDPA-treated substrates are used.

To check whether the HDPA treatment of the substrate removes deep traps in PVK-1.63, a rate-equation model was used to fit the tr-PL using three defects with variable trap depths.^13^Note S1 in the Supporting Information provides the details of the numerical model. Figure S9a shows the fitted differential decay time, and the corresponding fit parameters are shown in Table S1. For PVK-1.63 on neat glass, the three trap energies are 10, 120, and 310 meV relative to the valence or conduction band. Note that these defects can be donor-like or acceptor-like defects and cannot be distinguished by fitting as the perovskite is considered to be an intrinsic semiconductor. Using HDPA-functionalization, the deep defect at 310 meV in PVK-1.63 disappeared and has become shallow, with the new trap energies being 10, 80, and 10 meV (Table S1). This demonstrates that the long and continuously increasing decay times for PVK-1.63 films deposited on HDPA-treated substrates are dominated by shallow defects and not limited by deep defects as for the neat glass substrates.

It has been demonstrated that molecules with phosphonic acid anchoring groups added to the perovskite bulk also reduce nonradiative losses and enhance the *V*_OC_.^22−24^ To investigate whether loosely bound HDPA molecules on the substrate improve the perovskite bulk properties, glass/HDPA substrates were washed with 4:1 (v:v) of DMF:DMSO to simulate the deposition of the perovskite precursor before PVK-1.63 was deposited. The QFLS and τ_diff_ measured on the PVK-1.63 films were identical between the washed and the unwashed substrates (Figure S10), suggesting that loosely bound HDPA molecules on the substrate do not affect the perovskite PL decay and QFLS. The concentration of HDPA on the substrate surface will therefore most likely not affect the nonradiative losses of the perovskite, as has been demonstrated before for 2PACz.^25^ Following the procedure of Zheng et al.,^26^ HDPA was also added to the PVK-1.63 precursor solution (0.5 mg mL^–1^) as a bulk additive and was deposited on neat glass. The τ_diff_ of the PVK-1.63 films with HDPA added to the bulk on neat glass is longer than that for PVK-1.63 films without bulk additive, but shorter than that for PVK-1.63 on glass/HDPA (Figure S10a). In accordance, the QFLS of 1217 ± 7 meV measured by ss-PL for PVK-1.63 on glass increased by about ∼75 meV when HDPA was used as a bulk additive. This demonstrates that HDPA can improve the quality of the substrate/perovskite interface when used as a bulk additive and we conjecture that HDPA molecules migrate to the bottom and top interface of the perovskite to passivate interfacial defect states.^26^ From depth-profiling X-ray photoelectron spectroscopy (XPS), it was not feasible to spatially locate HDPA in the perovskite layer, because no phosphorus signal was detected for PVK-1.63 on glass/HDPA or for PVK-1.63 on glass with HDPA bulk additive (Figure S11).

*Extending HDPA Treatment to Different Perovskites*. Deposition of PVK-1.63 films on HDPA-functionalized glass or glass/ITO resulted in the highest QFLS and the longest differential decay time, consistent with the presence of shallow traps and the absence of deep traps. To demonstrate the general applicability of substrate functionalization with HDPA to increase the QFLS and more closely approach the detailed-balance limit for neat perovskite films, we extended the ss-PL and tr-PL experiments to perovskite compositions with different bandgaps (Figure S1). Next to PVK-1.63, we also studied Cs_0.1_FA_0.6_MA_0.3_Pb_0.5_Sn_0.5_I_3_ (*E*_g_ = 1.26 eV, PVK-1.26), Cs_0.2_FA_0.8_Pb(I_0.6_Br_0.4_)_3_ (*E*_g_ = 1.77 eV, PVK-1.77), and FAPbBr_3_ (*E*_g_ = 2.28 eV, PVK-2.28) perovskite semiconductors on glass, glass/HDPA, and glass/ITO/HDPA. We have chosen this set of perovskites for their diverse bandgaps, varying chemical compositions, and different fabrication procedures to highlight the diversity of perovskites for which HDPA treatment of the substrate reduces nonradiative recombination losses. Current density–voltage (*J*–*V*) characteristics of PSCs using these four perovskite compositions in p-i-n configurations show good device performance (Figure S12) and verify the high quality of the perovskite compositions and deposition procedures.

Each perovskite shows a very similar crystal structure for films deposited on neat glass or glass/HDPA substrates with no Bragg peaks appearing or disappearing in the XRD (Figure S13). The absence of significant changes indicates that deposition on HDPA does not strongly affect the perovskite crystal structure. Scanning electron microscopy (SEM) images also show similar grain appearances between glass and glass/HDPA (Figure S14), albeit with a slightly reduced grain size for perovskite films on glass/HDPA compared to that of the same perovskite on neat glass (Figure S15).

To investigate the nonradiative losses for the different perovskite compositions, tr-PL and absolute ss-PL were performed to determine the differential decay time and QFLS. For the PVK-1.26 films on neat glass, the QFLS was 895 ± 6 meV and showed no enhancement when the film was deposited on HDPA-functionalized substrates, i.e., 886 ± 10 meV on glass/HDPA and 896 ± 8 meV on glass/ITO/HDPA (Figure 3c). These values correspond to ∼89% of the radiative voltage limit for this perovskite composition (*qV*_OC_^rad^ = 991 meV). Similar values were obtained for PVK-1.26 on SAM-based HTLs such as Me-4PACz and 2PACz, but the QFLS is higher than that for PVK-1.26 on poly(3,4-ethylenedioxythiophene):poly(styrenesulfonate) (PEDOT:PSS) (867 ± 8 meV) which is often used in narrow-bandgap PSCs (Figure S16b). The reason that PVK-1.26 deposition on HDPA did not result in a higher QFLS compared to glass can be related to the known differences in crystallization of Pb–Sn-based and Pb-based perovskites,^27^ or to energetic differences associated with the narrow bandgap. Due to limitations of the experimental setup, tr-PL measurements for PVK-1.26 were not possible (see Experimental Section, Supporting Information).

**Figure 3:**
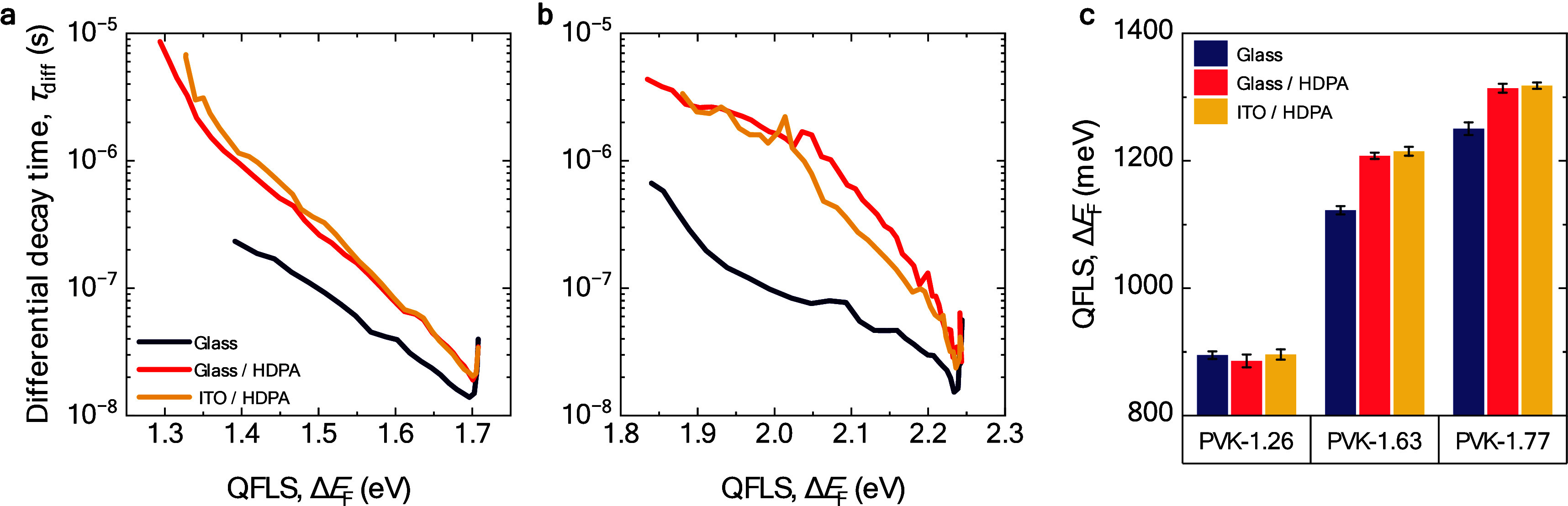
Transient and steady-state photoluminescence of various perovskites on different substrates. (a) Differential PL decay time against QFLS for PVK-1.77 deposited on glass, glass/HDPA, or glass/ITO/HDPA substrates. (b) Same for PVK-2.28 eV. (c) QFLS determined from the active side of PVK-1.26, PVK-1.63, and PVK-1.77 perovskite films deposited on glass, glass/HDPA, and glass/ITO/HDPA substrates.

For PVK-1.77 on glass/HDPA and glass/ITO/HDPA, the τ_diff_ determined from the tr-PL decay is longer for every QFLS than on neat glass and reaches up to ∼9 μs (Figure 3a). The fitting of the differential decay time of PVK-1.77 on neat glass and glass/HDPA shows that one defect becomes shallower, from 240 to 140 meV, when HDPA was used (Figure S9b and Table S2). This result corroborates the enhanced QFLS from absolute ss-PL, where PVK-1.77 on HDPA-treated substrates results in a ∼66 meV gain, reaching a QFLS of 1318 ± 5 meV, which is ∼160 meV below and corresponds to 89% of the radiative voltage limit for this perovskite composition (*qV*_OC_^rad^ = 1484 meV) (Figure 3c).

For the PVK-2.28 perovskite, an enhanced PL decay time for all QFLS of glass/HDPA and for glass/ITO/HDPA substrates is observed, showing a detectable τ_diff_ of ∼4 μs, compared to ∼700 ns for PVK-2.28 on glass (Figure 3b). Fitting the decay time for PVK-2.28 reveals that HDPA treatment of glass removes one deep defect from 340 to 10 meV (Figure S9c and Table S3). Interestingly, unlike the PVK-1.63 and PVK-1.77 films, for the PVK-2.28 on HDPA, the τ_diff_ starts to tail off at QFLS < 2 eV to a constant decay time corresponding to a Shockley–Read–Hall (SRH) recombination process which can be ascribed to the deeper defect still present at 130 meV (Table S3).^21^ Due to experimental limitations, determining the QFLS from ss-PL was not feasible for PVK-2.28 (see Experimental Section, Supporting Information).

To substantiate the result that functionalization of the substrate by HDPA results in the most defect-free perovskite semiconductors, the four different bandgap perovskites were deposited on glass/ITO substrates covered with common HTLs, i.e., PEDOT:PSS, 2PACz, and Me-4PACz. For all compositions, the HDPA-treated substrates resulted in the lowest nonradiative losses as inferred from the highest QFLS (Figure S16) and the longest PL differential decay times (Figures S17 and S18). This demonstrates that common HTLs induce (significant) losses compared to the perovskite films on HDPA and suggests that the HTL/perovskite interfaces limit the device performance. Designing new SAMs with charge selectivity that incorporates HDPA-like characteristics would provide a promising pathway to further reduce voltage losses in perovskite solar cells.

Summarizing, by studying the radiative recombination and the nonradiative losses of four perovskite compositions with different bandgaps (1.26, 1.63, 1.77, and 2.28 eV), we found that depositing the perovskite films on glass or ITO-covered glass substrates grafted with 1,6-hexylenediphosphonic acid (HDPA) generally reduces the nonradiative losses compared to neat glass and a range of other surface modifications tested. Modeling of the tr-PL reveals that HDPA substrate functionalization removes deep defects in the perovskite bulk or at the substrate/perovskite interface. Perovskite films on HDPA gave rise to a QFLS that best approached the radiative voltage limit of the neat defect-free perovskite film. We tentatively ascribe the reduced nonradiative losses to improved perovskite crystallization (leading to a more defect-free film) or to reduced surface recombination on HDPA-functionalized substrates. Hence, the use of glass or glass/ITO substrates functionalized with HDPA is a suitable method to approach the PL properties of defect-free perovskite thin films. Perovskite layers deposited on HDPA thus provide an appropriate reference system (compared to neat glass, quartz, or glass/ITO) to quantify, via PL spectroscopy, the nonradiative recombination losses caused by charge-selective transport layers below or atop the perovskite film and to measure the effect of bulk or surface passivation strategies. Finally, our findings accentuate that glass and ITO-covered glass substrates are not suitable as reference substrates for PL techniques involving metal-halide perovskites, because they create interface or bulk defects.

## Supporting Information Available

Experimental section and EQE spectra, ionization energies and QFLS, SFE, film thickness, average roughness, XRD, decay time vs QFLS, XPS, *J*–*V* plots, SEM images, grain size distribution histograms, tr-PL traces, tables of fitting parameters, and note about numerical simulations (PDF)
