# Supplementary material for: Functionalized Substrates for Reduced Nonradiative Recombination in Metal-Halide Perovskites
Source: J Phys Chem Lett. 2024 Dec 30;16(1):372–7. doi: 10.1021/acs.jpclett.4c03307 (PMC11726797; doi:10.1021/acs.jpclett.4c03307)
Supplement: Supplementary file 1 — jz4c03307_si_001.pdf [file jz4c03307_si_001.pdf]

Supporting information to

Functionalized substrates for reduced nonradiative recombination in metal-halide perovskites

Guus J. W. Aalbers,[1] Willemijn H. M. Remmerswaal,[1] Ralph H. C. van den Heuvel,[1] Laura Bellini,[1] Lana M. Kessels,[1] Christ H. L. Weijtens,[1] Nick R. M. Schipper,[1] Martijn M. Wienk,[1] & René A. J. Janssen*[1,2]

[1] *Molecular Materials and Nanosystems & Institute for Complex Molecular Systems, Eindhoven University of Technology, P.O. Box 513, 5600 MB Eindhoven, The Netherlands.*

[2] *Dutch Institute for Fundamental Energy Research, De Zaale 20, 5612 AJ Eindhoven, The Netherlands*

* *E-mail: r.a.j.janssen@tue.nl*

Contents

Experimental Section	page 2 − 13
Figures S1 – S12	page 14 − 25
Tables S1 – S3	page 26
Supplementary Notes	page 27
Supplementary References	page 28

Experimental Section

Materials

Lead iodide (PbI_2, 99.99% trace metal basis), lead bromide ($PbBr_2$, >98%), and guanidine thiocyanate (GuaSCN, >99.0%) were purchased from TCI Chemicals. Methylammonium bromide (MABr, >99.99%), methylammonium iodide (MAI, >99.99%), formamidinium iodide (FAI, >99.99%), formamidinium bromide (FABr, >99.99%), formamidinium thiocyanate (FASCN, >99%), and propane-1,3-diammonium iodide ($PDAI_2$) were purchased from Greatcell Solar Materials. Anhydrous tin(II) iodide beads (SnI_2, 99.99% trace metals basis), tin(II) fluoride (SnF_2, 99%), anhydrous cesium iodide beads (CsI, 99.999% trace metals basis), and glycine hydrochloride (GlyHCl, ≥99% (HPLC)) were purchased from Sigma Aldrich. [2-(9*H*-carbazol-9-yl)ethyl] phosphonic acid (2PACz, >98.0%), [4-(3,6-dimethyl-9*H*-carbazol-9-yl)butyl] phosphonic acid (Me-4PACz, >98%), 2-carboxyethyl phosphonic acid (CEPA, >98%), 1,6-hexylene diphosphonic acid (HDPA, >98%), and C_{60}-fused N-methylpyrrolidine-*m*-C_{12}-phenyl (CMC, >97.0%) were purchased from TCI Chemicals. Phenylphosphonic acid (PPA, 98%) and 4-aminobutyl phosphonic acid (ABPA, 97.5%) were purchased from Janssen Chimica. Poly[bis(4-phenyl)(2,4,6-trimethylphenyl)amine] (PTAA) was purchased from Solaris Chem. Poly(3,4-ethylenedioxythiophene) polystyrene sulfonate dispersion (PEDOT:PSS, P VP Al 4083) was purchased from Heraeus Clevios. [6,6]-phenyl-C_{61}-butyric acid methyl ester (PCBM, 99%) was purchased from Solenne BV. Indene-C_{60} bisadduct (ICBA) and bathocuproine (BCP, >99.5%) were purchased from Lumtec. Dimethylformamide (DMF, anhydrous 99.8%), dimethyl sulfoxide (DMSO, anhydrous 99.9%), anisole

(anhydrous 99.7%), propan-2-ol (IPA, anhydrous 99.95%), chlorobenzene (CB, anhydrous 99.8%), chloroform (CF, anhydrous >99%), and aluminum oxide (Al_2O_3) nanoparticle dispersion (20 wt.% in IPA) were purchased from Sigma Aldrich. Ethanol (>95%) and dodecyl sodium sulfate (99%) were purchased from Acros Organics. Nickel oxide (NiO_x) nanoparticle ink was purchased from Avantama. C_{60} fullerene (99.99%) was purchased from SES Research. Glass substrates were purchased from Schott (BOROFLOAT®) and indium-tin-oxide-covered glass substrates were purchased from Naranjo B.V.

Solution preparation

All solutions were prepared in a nitrogen environment.

PVK-1.63 precursor solution

Triple-cation $Cs_{0.05}(FA_{0.83}MA_{0.17})_{0.95}Pb(I_{0.83}Br_{0.17})_3$ perovskite (PVK-1.63) solutions were prepared by mixing $FAPbI_3$ and $MAPbBr_3$ (1.5 mol L^{-1}) in DMF:DMSO 4:1 (v:v) solutions. First, $FAPbI_3$ solution was made by dissolving PbI_2 (574 mg, 1.25 mmol) and FAI (177.4 mg, 1.03 mmol) in DMF (664 µL) and DMSO (166 µL). The $MAPbBr_3$ solution was prepared by dissolving $PbBr_2$ (93.6 mg, 0.26 mmol) and MABr (24.1 mg, 0.22 mmol) in DMF (136 µL) and DMSO (34 µL). $FAPbI_3$ and $MAPbBr_3$ were allowed to fully dissolve under stirring at 60 °C for 15 minutes. Then, the $FAPbI_3$ and $MAPbBr_3$ solutions were mixed in 83:17 (v:v) to achieve the desired I:Br ratio. Then, CsI solution (50 µL, 1.5 mol L^{-1} in DMSO) was added to the FAMA mixture to yield a CsFAMA solution and was allowed to fully mix under stirring at 60 °C for 15 minutes. This results in a PVK-1.63 precursor solution ready for spin coating.

PVK-1.26 precursor solution

Triple-cation lead-tin $Cs_{0.1}FA_{0.6}MA_{0.3}Pb_{0.5}Sn_{0.5}I_3$ perovskite (PVK-1.26) solutions were made by dissolving PbI_2 (0.9 mol L^{-1}), SnI_2 (0.9 mol L^{-1}), CsI (0.18 mol L^{-1}), FAI (1.08 mol L^{-1}), and MAI (0.54 mol L^{-1}) in a 3:1 DMF:DMSO (v:v) mixture. Additionally, SnF_2 (0.045 mol L^{-1}), GuaSCN (0.036 mol L^{-1}), and GlyHCl (0.036 mol L^{-1}) were added to the precursor stock before dissolving. The solutions were stirred for 40 minutes at 45 °C. After cooling to room temperature, the solutions were filtered using a 0.22 μm PTFE syringe filter.

PVK-1.77 precursor solution

Double-cation $Cs_{0.2}FA_{0.8}Pb(I_{0.6}Br_{0.4})_3$ perovskite (PVK-1.77) solutions were prepared by dissolving PbI_2 (221.3 mg, 0.48 mmol), $PbBr_2$ (264.3 mg, 0.72 mmol), FAI (165.1 mg, 0.96 mmol) and CsI (62.4 mg, 0.24 mmol) in 1 mL of DMF:DMSO 4:1 (v:v). The solution was stirred at 60 °C for 60 minutes and was cooled down to room temperature before use.

PVK-2.28 precursor solution

Wide-bandgap $FAPbBr_3$ perovskite (PVK-2.28) solutions were prepared by separately dissolving $PbBr_2$ (1.25 mol L^{-1}) in DMF:DMSO 9:1 (v:v) and FABr (0.49 mol L^{-1}) in IPA. The first was made by dissolving $PbBr_2$ (458.8 mg, 1.25 mmol) in DMF (900 μL) and DMSO (100 μL). For the latter, FABr (61.2 mg, 0.49 mmol) was dissolved in IPA and FASCN (1.26 mg, 2.5 mol% with respect to FABr) was added to the FABr solution as a bulk additive. The resulting $PbBr_2$ and FABr solutions were stirred overnight at 60 °C and cooled down to room temperature before use.

Solutions for functionalization, passivation, and charge transport

Solutions to functionalize glass and ITO-covered glass substrates were made by dissolving HDPA, CEPA, ABPA, or PPA in anhydrous ethanol (0.33 mg mL^{-1}) and sonicating for 30 minutes before spin coating. HTL solutions were prepared by dissolving 2PACz and Me-4PACz in anhydrous ethanol (0.33 mg mL^{-1}) and sonicating for 30 minutes before spin coating. PTAA was dissolved in toluene (3 mg mL^{-1}) and stirred overnight at 60 °C. The PEDOT:PSS dispersion was filtered using a 0.45 μm PVDF syringe filter before use.

The $PDAI_2$ top passivation solution (0.5 mg mL^{-1}) was prepared by dissolving $PDAI_2$ in IPA:CB 2:1 (v:v) and stirred overnight at 60 °C.

The solution-processed electron-transport layers (ETLs) were prepared by dissolving PCBM in CB at 15 mg mL^{-1}. The ternary fullerene mixture was prepared by dissolving PCBM, CMC, and ICBA in CF:CB 1:1 (v:v) at 20 mg mL^{-1}. To get the ternary mixture, equal volumes of each fullerene solution were mixed to yield the 1:1:1 (v:v:v) ternary blend and was filtered using a 0.22 μm PTFE syringe filter before use. All fullerene solutions were stirred overnight at 60 °C.

Sample fabrication for PL studies

Glass or ITO-covered glass substrates were cleaned by sonication in acetone for 15 minutes, followed by 30 s scrubbing and sonication with an aqueous solution of dodecyl sodium sulfate for 15 minutes. Then, the substrates were rinsed with deionized water for 15 minutes after they were sonicated in IPA for 15 minutes. The substrates were dried using a nitrogen gun and treated with UV-ozone for 30 minutes

prior to spin coating. Solutions (120 μL) to functionalize the surface with the materials shown in Figure 1 (phosphonic acid derivatives) were spin coated on the UV-ozone-treated glass or ITO-covered glass substrates for 30 s at 3000 rpm, followed by annealing at 100 °C for 10 minutes. To deposit PEDOT:PSS, a filtered PEDOT:PSS dispersion (150 μL) was spin coated in ambient conditions for 10 s at 500 rpm followed by 30 s at 4000 rpm (acceleration 20000 rpm s^{-1}). The substrate was then annealed for 20 minutes at 140 °C followed by a second annealing at 140 °C in a nitrogen-filled glovebox for 30 minutes. To deposit PTAA, 100 μL of PTAA solution was spin coated at 5800 rpm for 30 s (20000 rpm s^{-1}). The substrate was then annealed at 100 °C for 30 minutes.

On top of the (functionalized) glass and glass/ITO substrates perovskite films were deposited as follows:

PVK-1.63 films were deposited by spin coating the precursor (150 μL) for 35 s at 4000 rpm (acceleration of 800 rpm s^{-1}) on top of substrate. During spinning (after 20 s), the substrate was washed with 300 μL anisole as antisolvent. Then, the substrate was annealed at 100 °C for 30 minutes.

For PVK-1.26 films, 200 μL of the precursor solution was deposited on the substrate and the first spinning step was started for 10 s at 1000 rpm (acceleration 200 rpm s^{-1}), followed by a second step of 40 s at 4000 rpm (acceleration 1000 rpm s^{-1}). To quench the perovskite, 400 μL chlorobenzene was deposited on the film at 51 s after the start of the spinning. Next, the film was annealed at a hotplate of 65 °C for 10 minutes followed by 10 minutes at 100 °C.

For PVK-1.77 120 μL of the perovskite precursor solution was spin coated for 32 s at 4000 rpm (acceleration 1000 rpm s^{-1}), where 150 μL of anisole (antisolvent)

was dropped after 28 s from the start of spinning. The film was then annealed at 100 °C for 10 minutes.

PVK-2.28 films were fabricated via a two-step spin coating process in which the $PbBr_2$ solution (120 µL) was statically spin coated for 30 s at 3000 rpm (acceleration 20000 rpm s^{-1}) followed by 30 s at 3300 rpm (acceleration 20000 rpm s^{-1}). After 30 s from the start of the spinning, the FABr solution (100 µL) was dynamically deposited on top of the substrate. The resulting film was annealed at 100 °C for 30 minutes.

Solar cell fabrication

Pre-patterned glass/ITO substrates were cleaned as described for the samples for PL studies. The overlap between the bottom ITO and the top metal (Al or Ag) electrodes resulted in active solar cell areas of 0.09 and 0.16 cm^2.

For PVK-1.63 PSCs, a 2PACz solution (120 µL) was spin coated for 30 s at 3000 rpm, followed by annealing at 100 °C for 10 minutes. Then, the PVK-1.63 film was fabricated by spin coating the PVK-1.63 precursor (150 µL) for 35 s at 4000 rpm (acceleration of 800 rpm s^{-1}). During spinning (after 20 s), the substrate was washed with 300 µL anisole as antisolvent. Then, the substrate was annealed at 100 °C for 30 minutes. To complete the cell, fullerene C_{60} (20 nm), bathocuproine (BCP) (8 nm), and Ag (100 nm) were thermally evaporated under high vacuum.

For PVK-1.26 PSCs, the filtered PEDOT:PSS solution (150 µL) was spin coated in ambient conditions for 10 s at 500 rpm followed by 30 s at 4000 rpm (acceleration 20000 rpm s^{-1}). The substrate was then annealed for 20 minutes at 140 °C followed by a second annealing at 140 °C in a nitrogen-filled glovebox for 30 minutes. For the

spin coating of the PVK-1.26 films, 200 µL of the precursor solution was deposited on the substrate and the first spinning step was started for 10 s at 1000 rpm (acceleration 200 rpm s^{-1}), followed by a second step of 40 s at 4000 rpm (acceleration 1000 rpm s^{-1}). To quench the perovskite, 400 µL chlorobenzene was deposited on the film at 51 s after the start of the spinning. Next, the film was annealed on a hotplate at 65 °C for 10 minutes followed by 10 minutes at 100 °C. Finally, C_{60} (20 nm), BCP (8 nm), and Al (100 nm) were thermally evaporated under high vacuum to complete the device.

For PVK-1.77 devices, a NiO_x nanoparticle ink (120 µL) was spin-coated for 30 s at 3000 rpm (acceleration 1000 rpm s^{-1}) and used without annealing. Then, the Me-4PACz solution (120 µL) was spin coated on top of the NiO_x layer for 30 s at 3000 rpm (acceleration 1000 rpm s^{-1}) and annealed at 100 °C for 10 minutes. Subsequently, an Al_2O_3 nanoparticle dispersion (120 µL) was spin coated for 30 s at 4000 rpm (acceleration 2000 rpm s^{-1}), followed by annealing at 100 °C for 5 minutes. After letting the substrate cool to room temperature, 120 µL of PVK-1.77 perovskite precursor solution was spin coated for 32 s at 4000 rpm (acceleration 1000 rpm s^{-1}), where 150 µL of anisole (antisolvent) was dropped after 28 s from the start of spinning. The film was then annealed at 100 °C for 10 minutes. Subsequently, 150 µL of $PDAI_2$ solution was dynamically spin coated for 30 s at 4000 rpm (acceleration 20000 rpm s^{-1}) and annealed at 100 °C for 5 minutes. Finally, a PCBM solution (120 µL) was spin-coated for 30 s at 1000 rpm and used without further annealing. To complete the device, BCP (8 nm) and Ag (100 nm) were thermally evaporated under high vacuum.

For PVK-2.28 devices, a Me-4PACz solution with 10 mol% HDPA (120 µL) was spin coated for 30 s at 3000 rpm (acceleration 1000 rpm s^{-1}), followed by annealing at

100 °C for 10 minutes. PVK-2.28 films were fabricated via a two-step spin coating process in which the $PbBr_2$ solution (120 μL) was statically spin coated for 30 s at 3000 rpm (acceleration 20000 rpm s^{-1}) followed by 30 s at 3300 rpm (acceleration 20000 rpm s^{-1}). After 30 s from the start of the spinning, the FABr solution (100 μL) was dynamically deposited on top of the substrate. The resulting film was annealed at 100 °C for 30 minutes. Then, $PDAI_2$ (100 μL) was dynamically spin coated for 30 s at 4000 rpm. The substrate was annealed at 100 °C for 5 minutes. For the ETL, a ternary fullerene PCBM:ICBA:CMC (1:1:1) mixture solution (100 μL) was spin coated for 60 s at 1000 rpm and then annealed at 100 °C for 10 minutes, followed by thermal evaporation under high vacuum of LiF (1 nm) and Al (100 nm).

Quasi-Fermi level splitting

The QFLS was assessed through steady-state absolute photoluminescence (ss-PL) measurements. Samples were excited using a 455 nm Thorlabs M455F3 fiber-coupled LED. Samples were placed under an Avantes AvaSphere-30-REFL integrating sphere equipped with in-line filter holders for excitation light and emitted light, holding a 550 nm short-pass filter (Edmund Optics) and a 550 nm long-pass filter (Edmund Optics), respectively. The incident photon flux was adjusted to simulate AM1.5G conditions. The integrating sphere was connected to an Avantes AvaSpec-HSC1024X58TEC-EVO spectrometer (550−1100 nm) by an optical fiber. The setup was calibrated using an Avantes halogen lamp, yielding a spectral correction factor. Spectral photon fluxes ϕ_{PL} were obtained after a Jacobian transformation[1]. Using the nonlinear least squares fit method in MATLAB, the QFLS was determined from the ϕ_{PL}. The relation between QFLS and photon flux is defined as follows

$$\phi_{\mathrm{PL}}(E) = \frac{1}{4\pi^2\hbar^3c^2}\frac{a(E)E^2}{\exp\left(\frac{E-\Delta E_{\mathrm{F}}}{k_{\mathrm{B}}T}\right)-1} \tag{1}$$

where $a(E)$ is the photon energy-dependent absorptivity[2,3], is assumed to be unity for photon energies sufficiently larger than the optical bandgap. Each film or (partial) stack combination was measured on 3 spots on the same film and on multiple films, the QFLS values were averaged and the standard deviation was determined. Next to variations in QFLS for different spots on the sample, there are batch-to-batch variations to inevitable small differences in the composition of the precursor solutions and processing conditions during the investigations. From the available data the standard deviation between nominally identical samples is estimated to be 15 meV or less.

Transient photoluminescence (tr-PL)

A pulsed Continuum Surelite-II 10 Nd:YAG laser with a 355 nm wavelength output was used as the excitation source with a repetition rate of 10 Hz. The laser beam passed through an aperture and hit the sample normal to the surface with a circular spot diameter of 2 mm. The excitation fluence was around 9.55 μJ cm^{-2}, resulting in an average charge carrier concentration (Δn) in PVK-1.63 of 3.41 × 10^{17} cm^{-3}. The initial charge carrier concentration resulted in an initial quasi-Fermi level splitting ($\Delta E_{\mathrm{F}}^{\max}$) of 1.50 eV for PVK-1.63 and was calculated as

$$\Delta E_{\mathrm{F}}^{\max} = \frac{2k_{\mathrm{B}}T}{q}\ln\left(\frac{\Delta n}{n_i}\right) \tag{2}$$

Where k_{B} is the Boltzmann constant, T the temperature, q the elementary charge, Δn the average initial carrier concentration, and n_i is the intrinsic carrier concentration

which is assumed to be 8×10^4 cm^{-3}, 2×10^3 cm^{-3}, and 6×10^{-2} cm^{-3} for PVK-1.63, PVK-1.77, and PVK-2.28, respectively. The Fermi-level splitting *x*-axis scale was determined using the relation

$$\Delta E_{\mathrm{F}}(t) = \Delta E_{\mathrm{F}}^{\mathrm{max}} + \frac{k_{\mathrm{B}}T}{q}\ln[\phi_{\text{tr-PL}}(t)] \quad (3)$$

Where $\phi_{\text{tr-PL}}(t)$ is the tr-PL decay. So, for every order of magnitude decrease in tr-PL, the ΔE_{F} is lowered by ~60 meV.

The emitted PL signal was focused on an optical fiber and coupled into a Princeton Instruments SpectraPro 300 spectrometer, and the spectrally dispersed signals were analyzed using a Roper Scientific PI-MAX HQ intensified CCD camera (450−900 nm). To yield time-resolved PL spectra, the shutter functionality of the iCCD camera was used along with an external trigger from the laser to capture a PL spectrum at changing waiting times after the trigger.

Surface free energy (SFE)

The surface free energy was determined using the sessile drop technique in which 2 μL droplets of either ultrapure milli-Q water (5.49×10^{-5} μS cm^{-1}; polar solvent) or pure diiodomethane (dispersive solvent) were deposited on the sample. Contact angles were measured in triplicate using a DataPhysics OCA30 contact angle goniometer at three different places on the sample. The OCA30 SEcalc software was used to calculate the surface free energy (SFE) using the Owens, Wendt, Rabel, and Kaelble (OWRK) method.

Scanning electron microscopy

SEM images were collected with an FEI Quanta 3D FEG microscope (5 keV electron beam, secondary electron detector) and a PhenomProX (5 keV electron beam, secondary electron detector).

X-ray diffraction

XRD was recorded using a Bruker 2D phaser (Cu Kα radiation, $\lambda = 1.5406$ Å). Measurements were performed in the range of 10−40° with a step size of 0.01° and collection time of 0.2 s. A divergence slit of 0.6 mm and an anti-scatter screen of 0.5 mm were employed.

X-ray photoelectron spectroscopy

XPS measurements were performed using a Thermo-Scientific K-Alpha with a 180° double-focusing hemispherical analyzer and a 128-channel detector. Monochromatic Al Kα (1486.6 eV) radiation was used, and the X-ray spot size was 400 μm. The depth-profile measurements were performed in etching mode with an ion energy of 500 eV and low current (sputter rate estimate of 0.05 nm s^{-1}). Each etch cycle had a duration of 20 s and 120 total levels were measured.

Ultraviolet photoelectron spectroscopy

The ionization energy was determined from UPS measurements using a VG EscaLab II system with a He-I light source of 21.22 eV and a −6 V bias. The chamber was kept under a pressure of 10^{-8} Pa.

Current density – voltage characteristics

The cells were tested in a N_2-filled glove box at ambient temperature. To emulate approximately 100 mW cm^{-2} AM1.5G light, a tungsten halogen lamp in combination with a Schott GG385 UV filter, and Hoya LB120 daylight filter were used. Incident light was referenced using a Si photodiode. Shadow masks of 0.0676 or 0.1296 cm^2 were used to define the illuminated area of the solar cell. J–V characteristics were determined using a Keithley 2400 SMU. The J–V scan swept the applied voltage bias (without pre-biasing) from +1.5 to −0.5 V for a reverse scan, or from −0.5 to +1.5 V for a forward scan using a scan rate of 250 mV s^{-1}.

Supplementary Figures

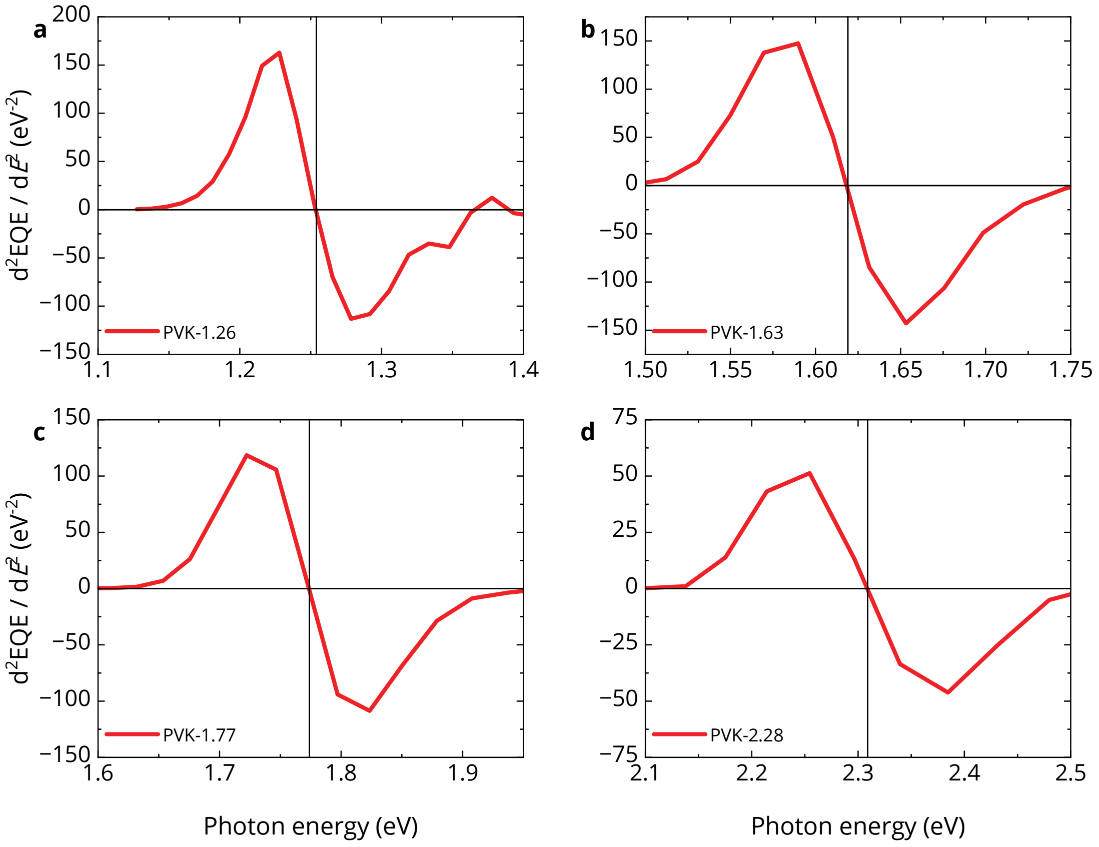

Figure S1. Second derivative of the EQE spectra (d^{2}EQE/dE^2) for different perovskite solar cells. (a) PVK-1.26. (b) PVK-1.63. (c) PVK-1.77. (d) PVK-2.28 perovskite. The optical bandgap is determined for d^{2}EQE/dE^2 = 0.

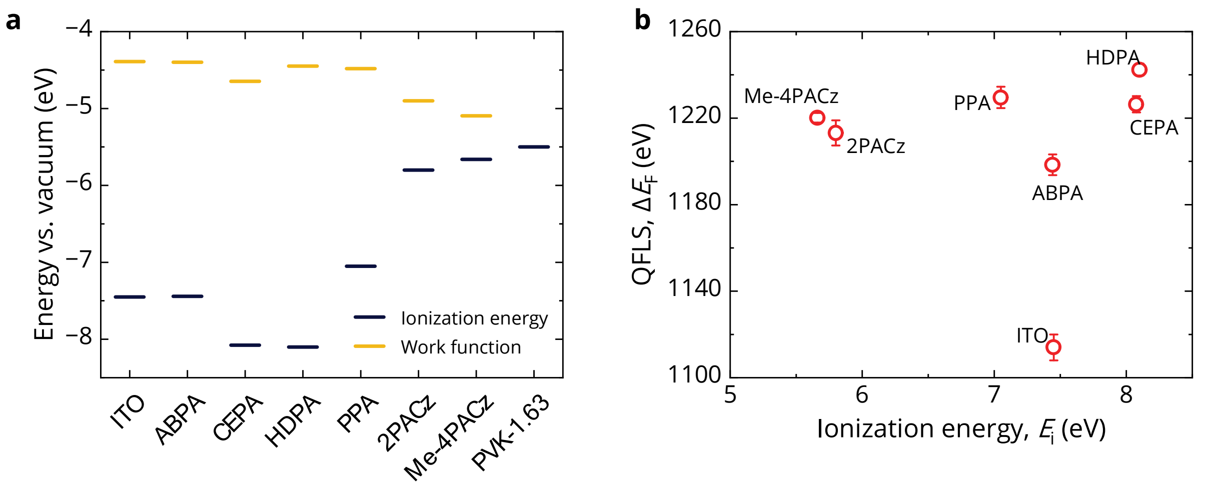

Figure S2. Glass/ITO substrates functionalized with ABPA, CEPA, HDPA, PPA, 2PACz, and Me-4PACz. (a) Ionization energies and work functions from ultraviolet photoelectron spectroscopy (UPS) of functionalized glass/ITO substrates and PVK-1.63 perovskite on glass/ITO. (b) QFLS of PVK-1.63 perovskite deposited on functionalized glass/ITO substrates versus their ionization energy shows no correlation.

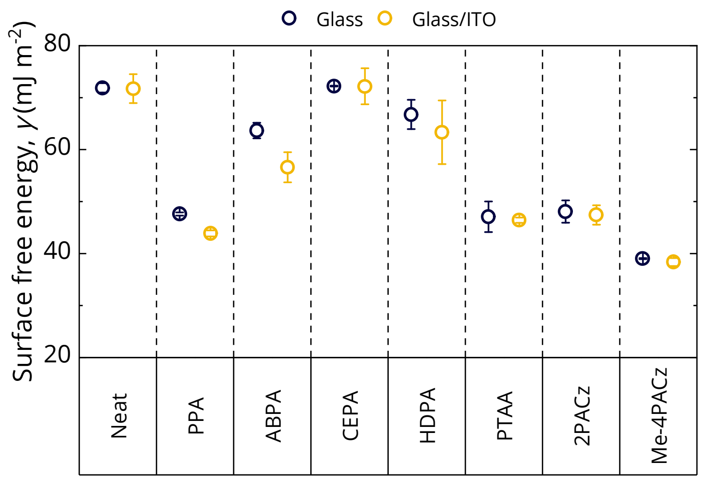

Figure S3. Surface free energy (SFE) of glass and glass/ITO substrates functionalized with different molecules.

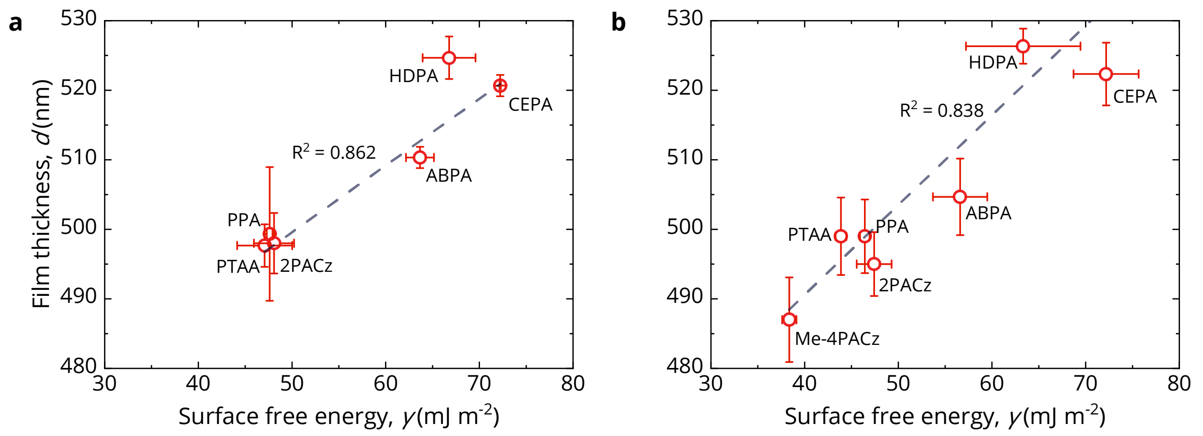

Figure S4. Film thickness of PVK-1.63 perovskite deposited on functionalized substrates versus the surface free energy (SFE) of the functionalized substrate. (a) On glass. (b**)** On glass/ITO. Dashed line is a linear fit with corresponding R-squared.

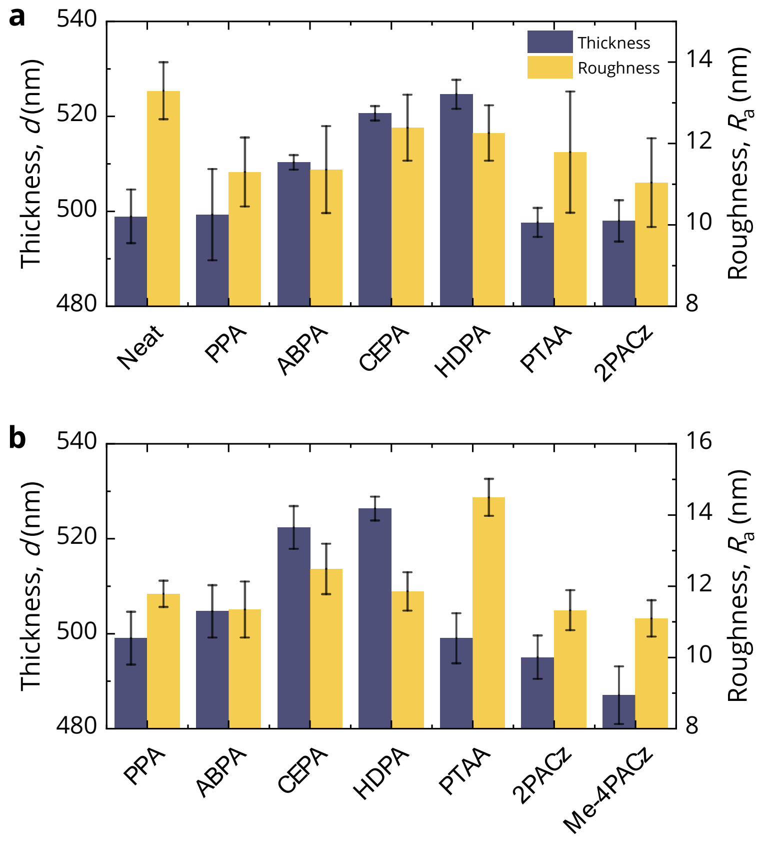

Figure S5. Film thickness and average roughness of PVK-1.63 perovskite deposited on functionalized (a) glass and (b) glass/ITO substrates.

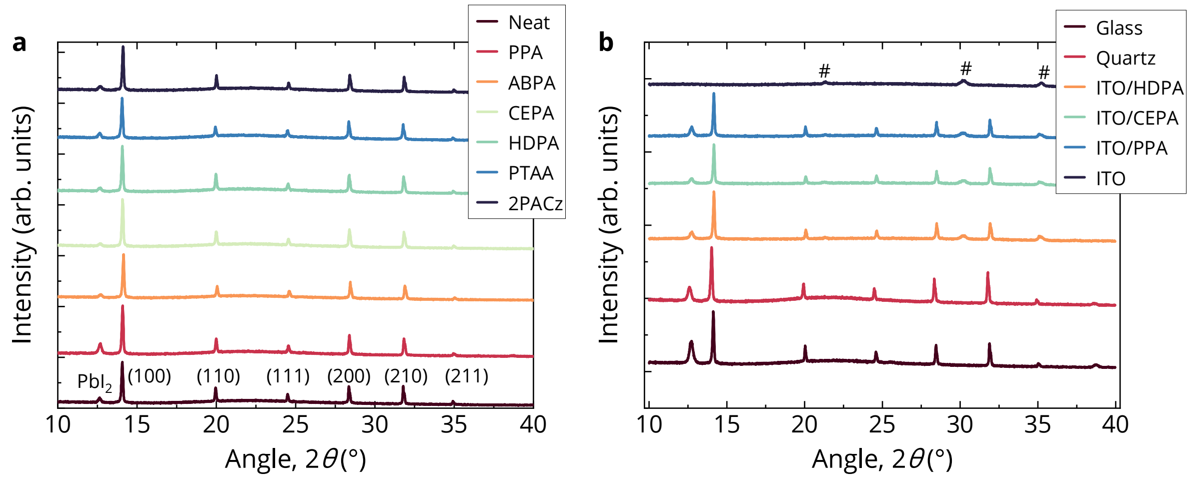

Figure S6. X-ray diffractograms (XRD) of PVK-1.63 perovskite deposited on substrates functionalized with different molecules. (a) On glass. (b) On glass/ITO. # denotes ITO diffraction peaks.

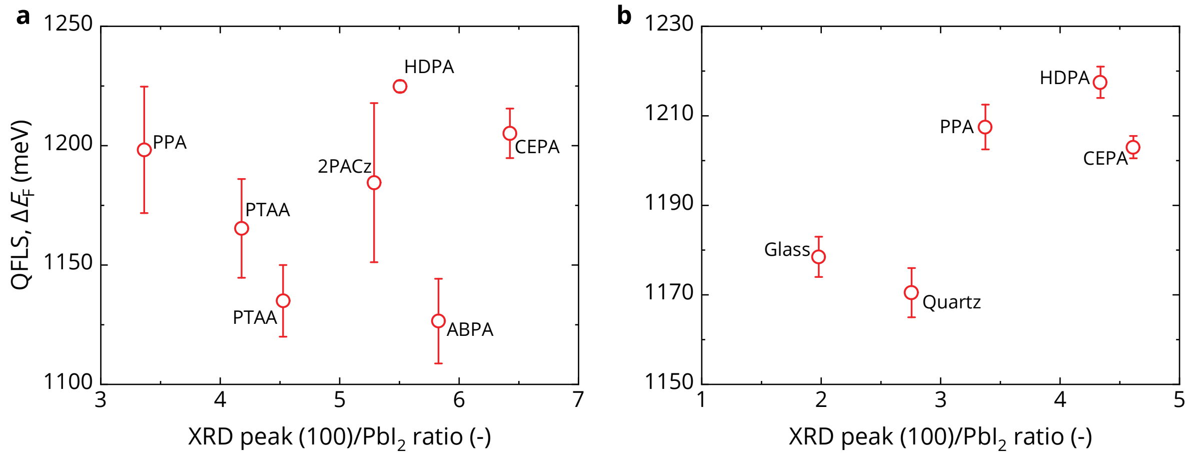

Figure S7. QFLS of PVK-1.63 films deposited on (a) glass and (b) glass/ITO substrates functionalized with different molecules versus the XRD Bragg peak ratio between (100) and PbI_2.

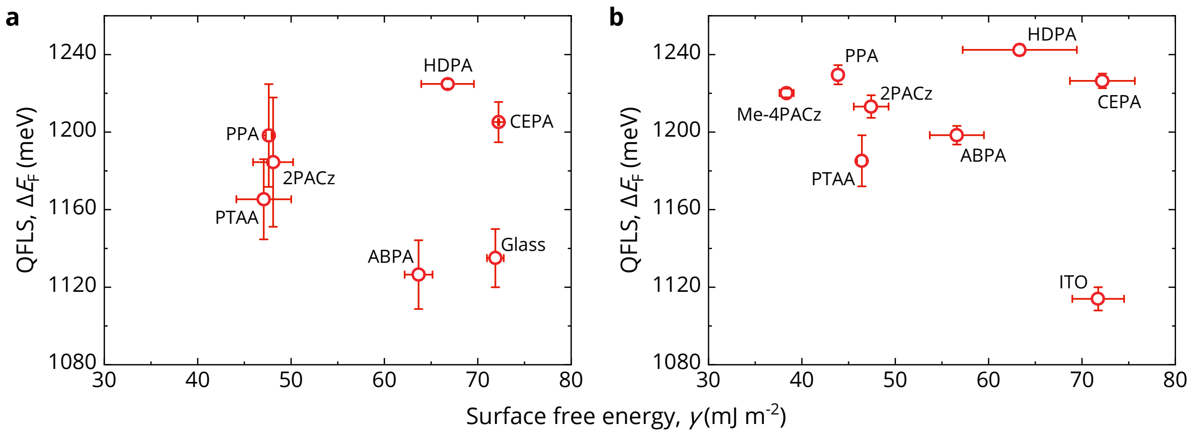

Figure S8. QFLS of PVK-1.63 perovskite deposited on functionalized substrates versus the surface free energy (SFE) of the functionalized substrate. (a) On glass. (b) On glass/ITO.

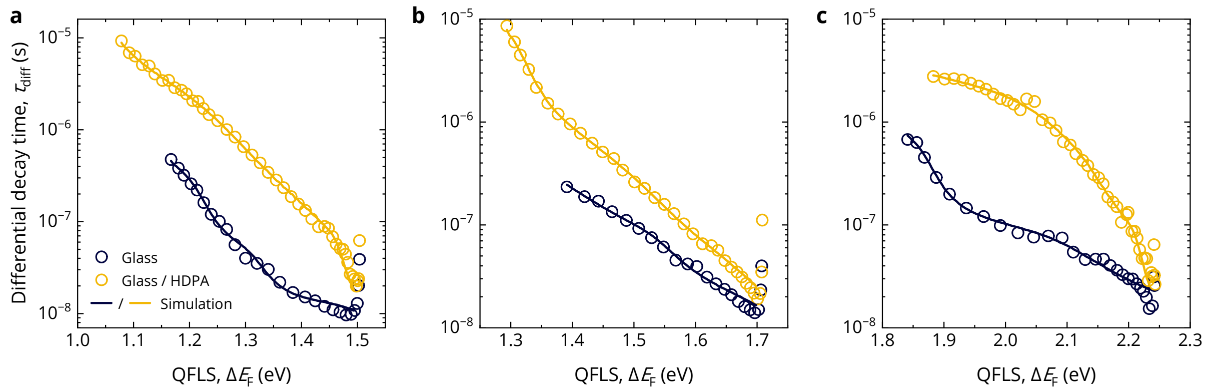

Figure S9. Differential decay time (τ_{diff}) as a function of the quasi-Fermi level splitting for perovskite films deposited on glass substrates with or without HDPA-treatment. Solid lines are fits using 3 defects. (a) PVK-1.63. (b) PVK-1.77. (c) PVK-2.28.

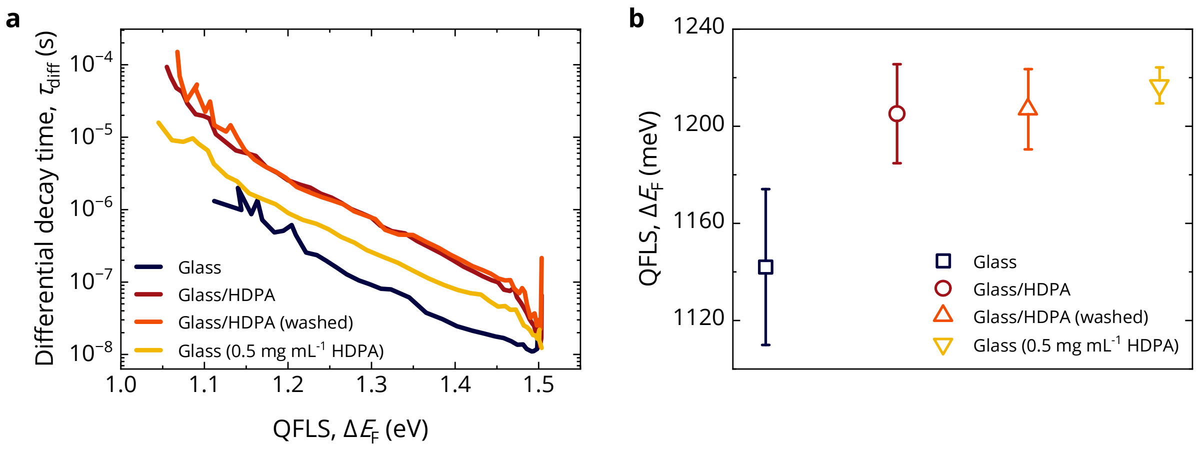

Figure S10. (a) Differential decay time (τ_{diff}) as a function of the quasi-Fermi level splitting for a PVK-1.63 perovskite films deposited on glass, glass/HDPA, glass/HDPA washed with 4:1 v:v DMF:DMSO, and on glass from a precursor solution with additional 0.5 mg mL^{-1} HDPA . (b) QFLS determined from absolute ss-PL for the same samples.

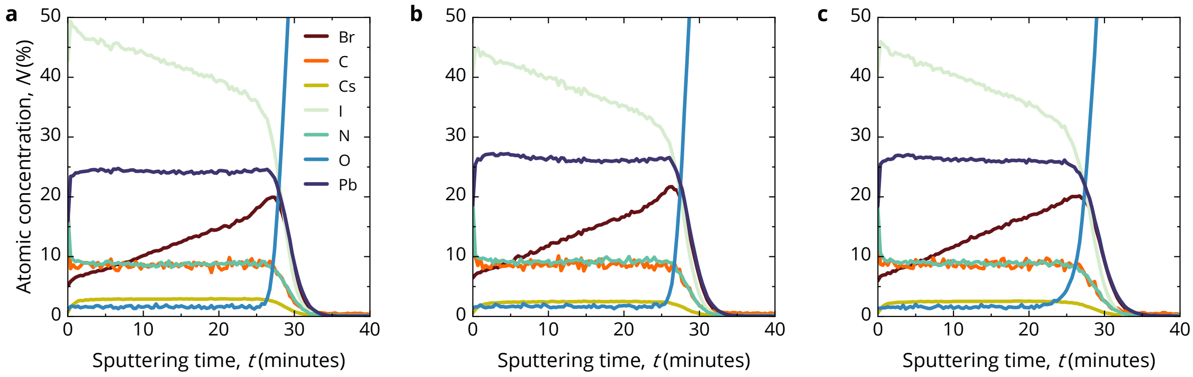

Figure S11. Depth-profiling X-ray photoelectron spectroscopy (XPS) of PVK-1.63 perovskite films. (a) Deposited on glass. (b) Deposited on glass/HDPA. (c) Deposited on glass from a precursor solution with additional 0.5 mg mL^{-1} HDPA.

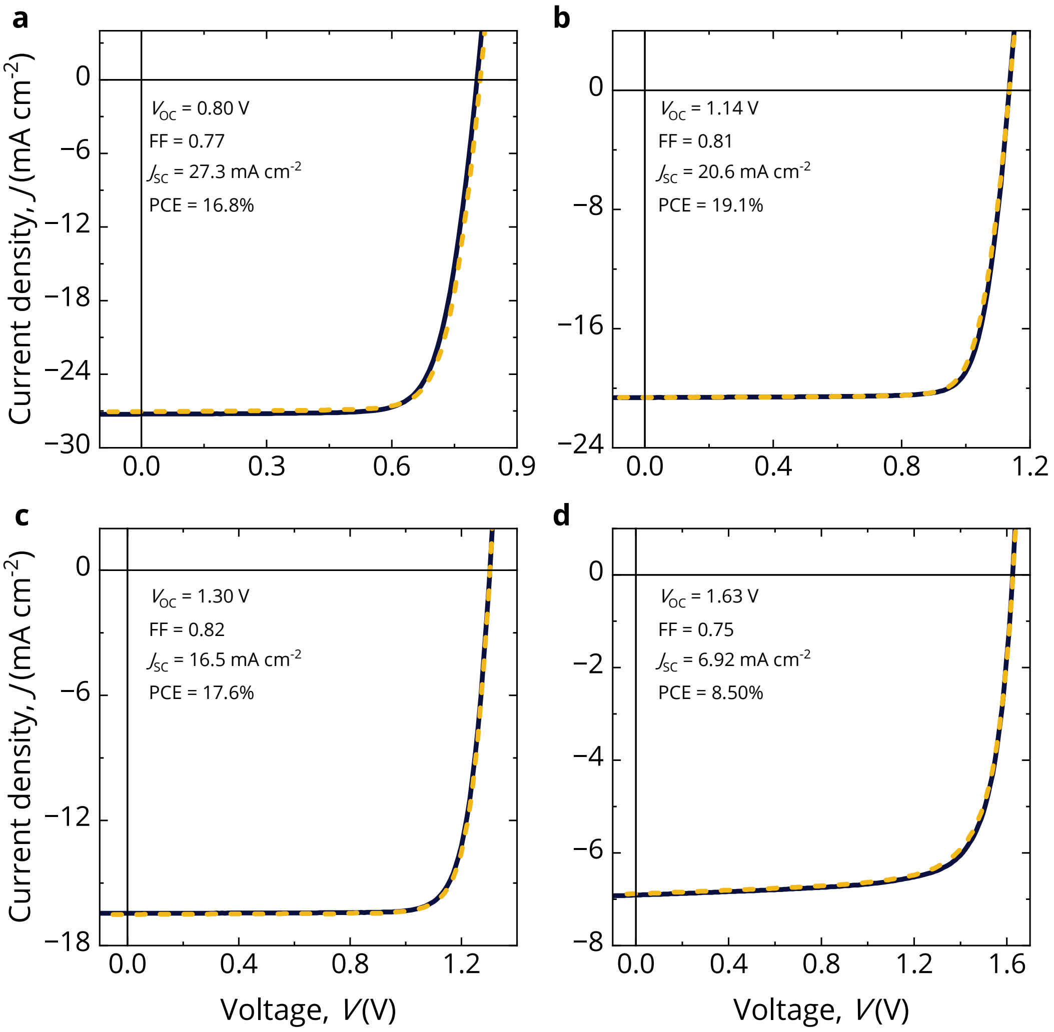

Figure S12. Current density – voltage (*J*-*V*) measurements in reverse (solid line) and forward (dashed line) scan directions of perovskite solar cells measured with simulated solar (AM1.5G, 100 mW cm^{-2}) illumination. (a) glass/ITO/PEDOT:PSS/ $Cs_{0.1}FA_{0.6}MA_{0.3}Pb_{0.5}Sn_{0.5}I_3/C_{60}$/BCP/Ag. (b) glass/ITO/2PACz/$Cs_{0.05}(FA_{0.83}MA_{0.17})_{0.95}$ $Pb(I_{0.83}Br_{0.17})_3/C_{60}$/BCP/Al. (c) glass/ITO/$NiO_x$ (np)/Me-4PACz/Al_2O_3 (np)/$Cs_{0.2}FA_{0.8}$ $Pb(I_{0.6}Br_{0.4})_3$ /$PDAI_2$/PCBM/BCP/Ag (np = nanoparticles). (d) glass/ITO/Me-4PACz + 10 mol% HDPA/$FAPbBr_3$/$PDAI_2$/PCBM:CMC:ICBA (1:1:1)/LiF/Al.

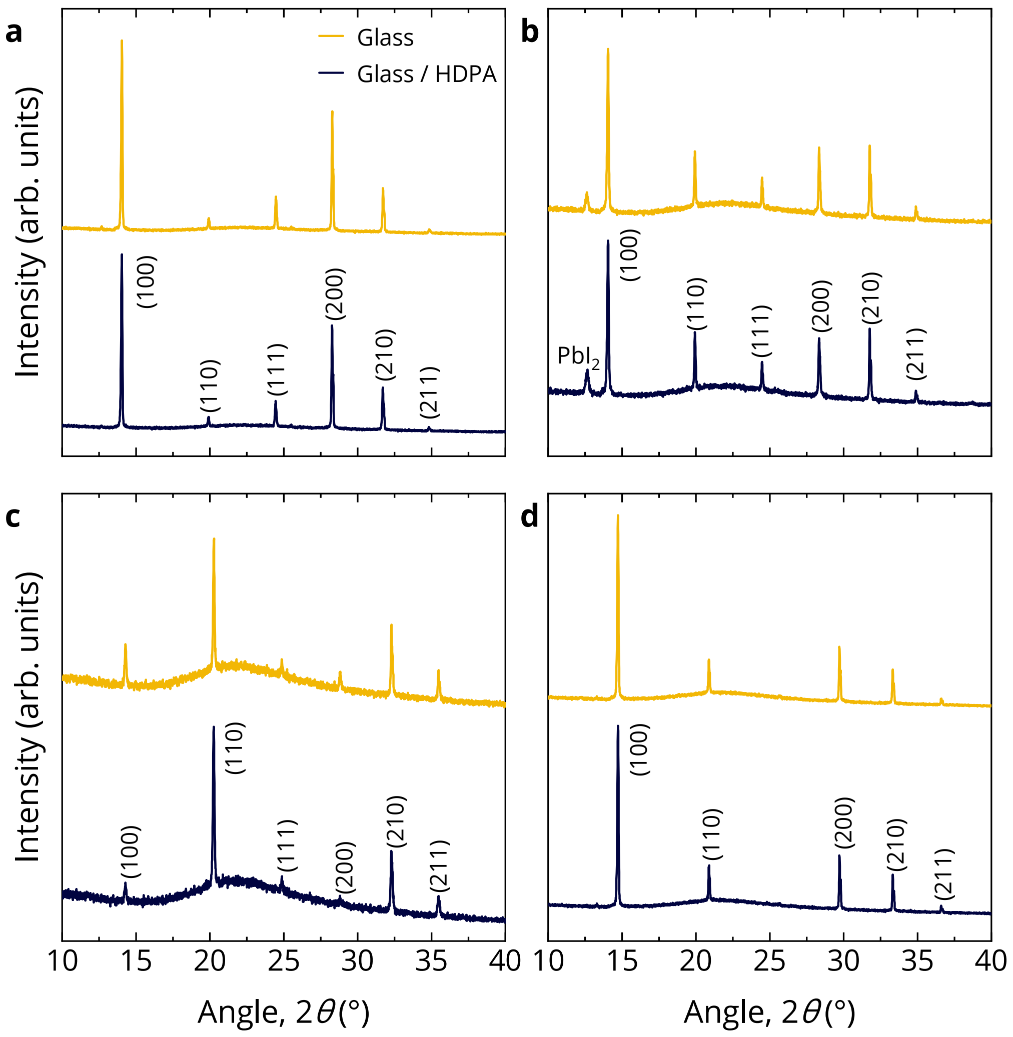

Figure S13. X-ray diffractograms. (a) PVK-1.26. (b) PVK-1.63. (c) PVK-1.77. (d) PVK-2.28 perovskite films deposited on glass or HDPA-functionalized glass.

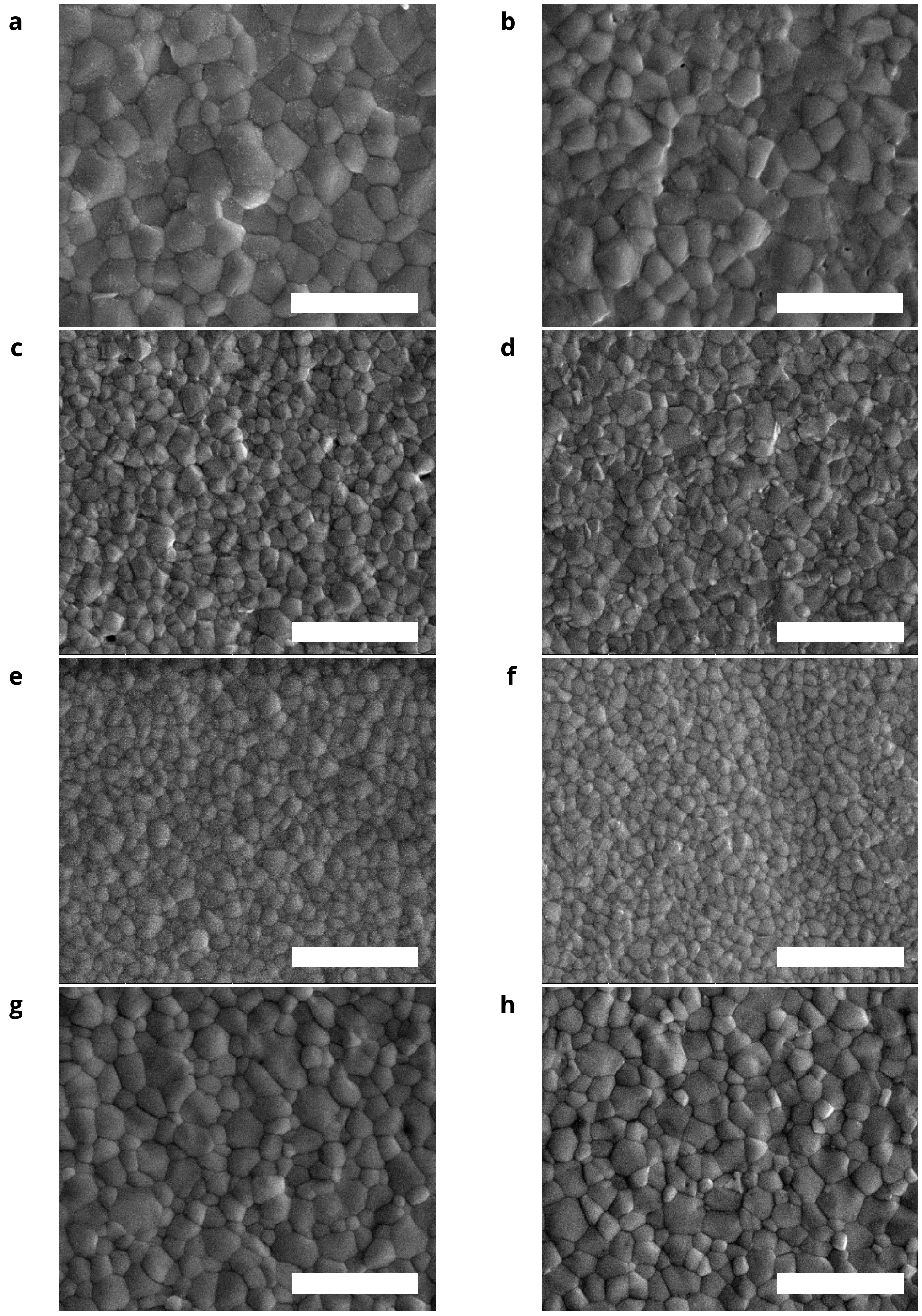

Figure S14. Scanning electron microscopy (SEM) images of perovskite films deposited on glass and HDPA-functionalized glass. (a) PVK-1.26 on glass. (b) PVK-1.26 on glass/HDPA. (c) PVK-1.63 on glass. (d) PVK-1.63 on glass/HDPA. (e) PVK-1.77 on glass. (f) PVK-1.77 on glass/HDPA. (g) PVK-2.28 on glass. (h) PVK-2.28 on glass/HDPA. Scale bars are 2 μm.

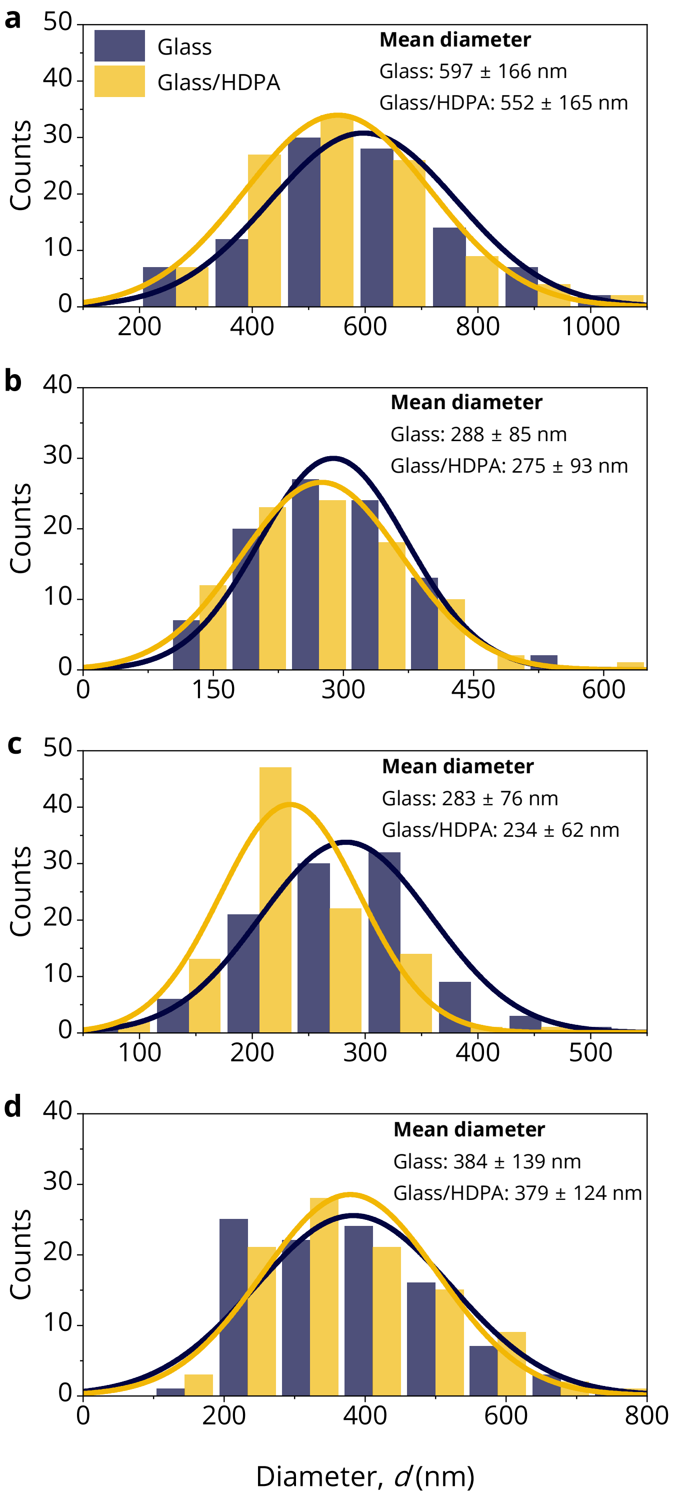

Figure S15. Grain size distribution histograms for (a) PVK-1.24, (b) PVK-1.63, (c) PVK-1.77, and (d) PVK-2.28 on glass or glass/HDPA substrates obtained from SEM images. Solid lines are Gaussian fits. N = 100.

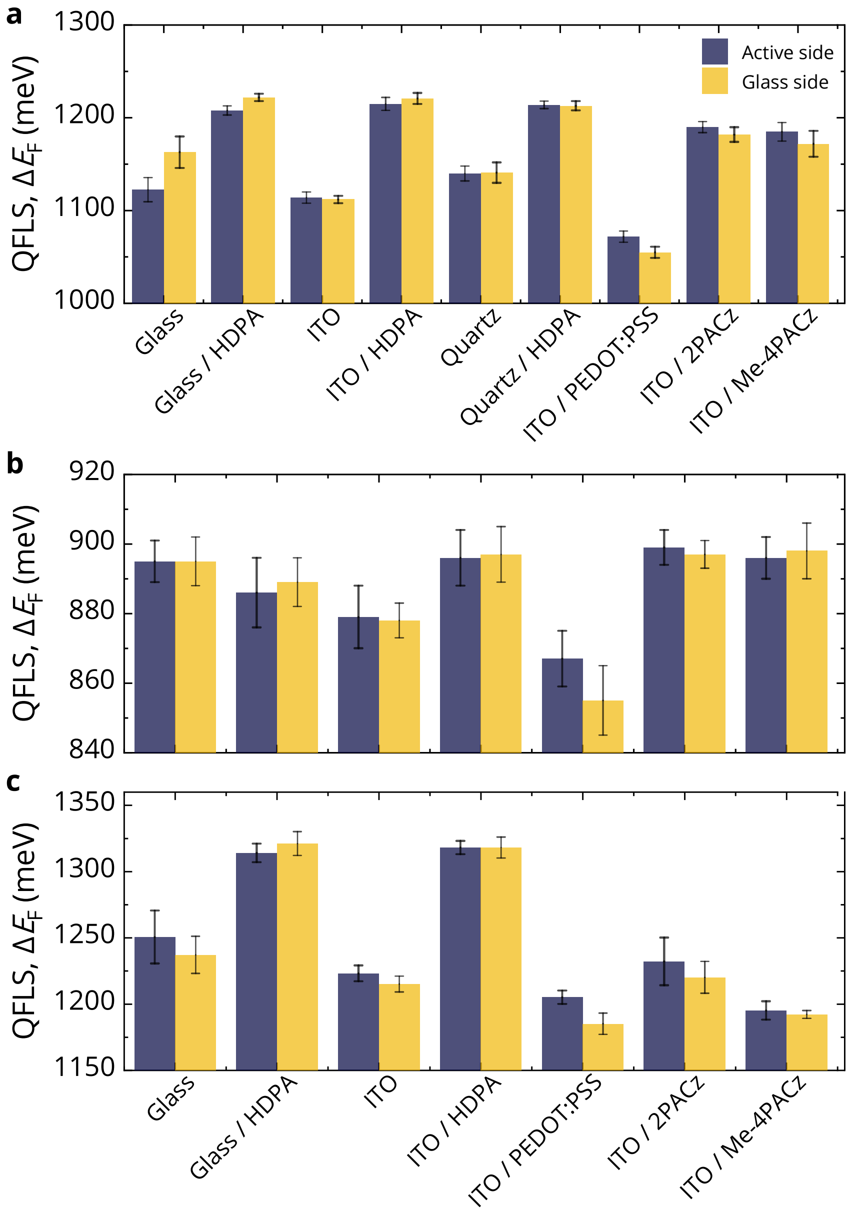

Figure S16. QFLS determined from absolute ss-PL measurements for perovskite films deposited on different substrates. (**a**) PVK-1.63. (**b**) PVK-1.26. (**c**) PVK-1.77. PL spectra were measured through the active side or the substrate side.

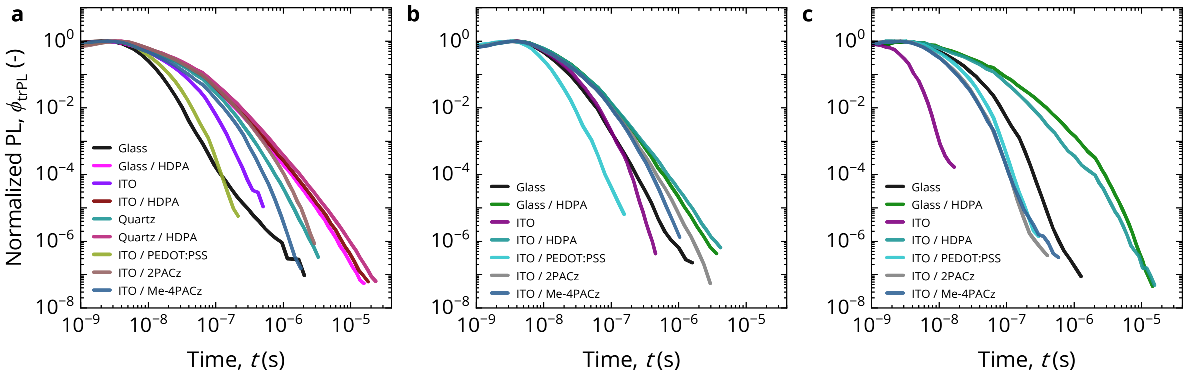

Figure S17. Transient photoluminescence (tr-PL) traces of perovskite films deposited on glass or glass/ITO substrates treated with various molecules or polymers. (a) PVK-1.63. (b) PVK-1.77. (c) PVK-2.28.

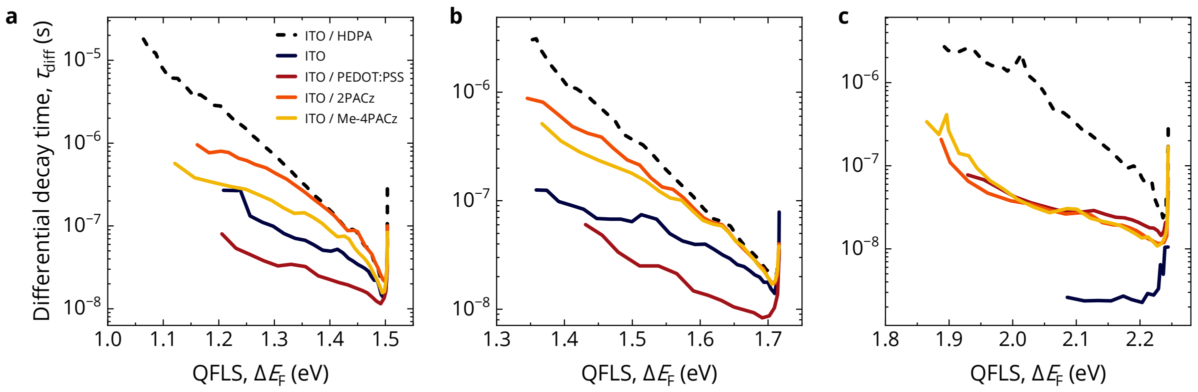

Figure S18. Differential decay time (τ_{diff}) as a function of the quasi-Fermi level splitting for perovskite films deposited on glass/ITO substrates treated with HDPA, or the HTLs PEDOT:PSS, 2PACz, or Me-4PACz. (a) PVK-1.63. (b) PVK-1.77. (c) PVK-2.28.

Table S1. Fitting parameters of three-defect numerical fit of PVK-1.63

	Trap #	k_{rad} [cm^6 s^{-1}]	N_t [cm^{-3}]	E_t [eV]	β_n [cm^3 s^{-1}]	β_p [cm^3 s^{-1}]	e_n [cm^3 s^{-1}]	e_p [cm^3 s^{-1}]
glass	1	9.00×10^{-11}	1.35×10^{18}	1.62	9.76×10^{-12}	1.43×10^{-10}	1.47×10^{7}	1.92×10^{-19}
	2	9.00×10^{-11}	1.25×10^{18}	1.51	1.52×10^{-11}	5.45×10^{-11}	3.33×10^{5}	5.05×10^{-18}
	3	9.00×10^{-11}	1.07×10^{18}	1.32	1.00×10^{-10}	2.47×10^{-11}	1.25×10^{3}	4.02×10^{-15}
HDPA	1	9.00×10^{-11}	1.29×10^{18}	1.62	5.16×10^{-12}	2.73×10^{-10}	7.21×10^{6}	3.96×10^{-19}
	2	9.00×10^{-11}	1.25×10^{18}	1.55	6.12×10^{-13}	3.61×10^{-10}	6.18×10^{4}	7.25×10^{-18}
	3	9.00×10^{-11}	1.23×10^{18}	1.62	5.95×10^{-11}	1.71×10^{-11}	8.95×10^{7}	2.31×10^{-20}

Table S2. Fitting parameters of three−defect numerical fit of PVK-1.77

	Trap #	k_{rad} [cm^6 s^{-1}]	N_t [cm^{-3}]	E_t [eV]	β_n [cm^3 s^{-1}]	β_p [cm^3 s^{-1}]	e_n [cm^3 s^{-1}]	e_p [cm^3 s^{-1}]
glass	1	8.37×10^{-11}	1.38×10^{18}	1.76	1.09×10^{-11}	1.10×10^{-10}	1.64×10^{7}	6.61×10^{-22}
	2	8.37×10^{-11}	1.22×10^{18}	1.76	2.92×10^{-12}	9.26×10^{-11}	4.40×10^{6}	5.56×10^{-22}
	3	8.37×10^{-11}	1.04×10^{18}	1.53	3.64×10^{-11}	7.30×10^{-11}	7.71×10^{3}	3.11×10^{-18}
HDPA	1	8.37×10^{-11}	1.65×10^{18}	1.76	1.74×10^{-12}	1.42×10^{-10}	2.62×10^{6}	8.52×10^{-22}
	2	8.37×10^{-11}	1.23×10^{18}	1.76	6.24×10^{-12}	1.60×10^{-10}	9.38×10^{6}	9.61×10^{-22}
	3	8.37×10^{-11}	1.10×10^{18}	1.63	3.56×10^{-12}	8.40×10^{-11}	3.80×10^{4}	7.11×10^{-20}

Table S3. Fitting parameters of three-defect numerical fit of PVK-2.28

	Trap #	k_{rad} [cm^6 s^{-1}]	N_t [cm^{-3}]	E_t [eV]	β_n [cm^3 s^{-1}]	β_p [cm^3 s^{-1}]	e_n [cm^3 s^{-1}]	e_p [cm^3 s^{-1}]
glass	1	5.00×10^{-11}	1.32×10^{18}	2.27	1.29×10^{-11}	1.27×10^{-10}	1.94×10^{7}	2.06×10^{-30}
	2	5.00×10^{-11}	1.33×10^{18}	2.13	7.30×10^{-12}	8.19×10^{-11}	5.66×10^{4}	2.58×10^{-28}
	3	5.00×10^{-11}	1.03×10^{18}	1.94	1.96×10^{-11}	3.20×10^{-11}	8.22×10^{1}	1.87×10^{-25}
HDPA	1	5.00×10^{-11}	1.40×10^{18}	2.27	4.09×10^{-12}	1.37×10^{-11}	6.15×10^{6}	2.23×10^{-31}
	2	5.00×10^{-11}	1.29×10^{18}	2.15	9.08×10^{-13}	4.84×10^{-10}	1.35×10^{4}	7.95×10^{-28}
	3	5.00×10^{-11}	1.03×10^{18}	2.27	5.77×10^{-11}	5.36×10^{-12}	8.68×10^{7}	8.71×10^{-32}

Supplementary Note S1

Numerical simulations for the transient PL (tr-PL) were performed using a MATLAB script based on the procedure and method of Yuan et al.[4] The script solves a coupled rate-equation model to fit the differential decay encompassing the time derivatives of the electron and hole concentration (n, p) and the occupied defect concentration for three defects.

$$\frac{\mathrm{d}n}{\mathrm{d}t} = -k_{\mathrm{rad}}(np - n_i^2) + G_{\mathrm{bias}} + \sum_{j=1}^{3} -\beta_n^{\mathrm{t}_j} n \left(N_{\mathrm{t}_j} - n_{\mathrm{t}_j}\right) + e_n^{\mathrm{t}_j} n_{\mathrm{t}_j} \tag{4}$$

$$\frac{\mathrm{d}p}{\mathrm{d}t} = -k_{\mathrm{rad}}(np - n_i^2) + G_{\mathrm{bias}} + \sum_{j=1}^{3} -\beta_p^{\mathrm{t}_j} p n_{\mathrm{t}_j} + e_p^{\mathrm{t}_j} \left(N_{\mathrm{t}_j} - n_{\mathrm{t}_j}\right) \tag{5}$$

$$\frac{\mathrm{d}n_{\mathrm{t}_j}}{\mathrm{d}t} = \beta_n^{\mathrm{t}_j} n \left(N_{\mathrm{t}_j} - n_{\mathrm{t}_j}\right) + \beta_p^{\mathrm{t}_j} pn - e_n^{\mathrm{t}_j} n_{\mathrm{t}_j} + e_p^{\mathrm{t}_j} \left(N_{\mathrm{t}_j} - n_{\mathrm{t}_j}\right) \tag{6}$$

Here, n and p are electron and hole densities, t is time, k_{rad} is radiative recombination coefficient, n_{i} is intrinsic carrier density, G_{bias} is external bias generation rate, and $\beta_{\mathrm{n}}^{\mathrm{t}}$ and $\beta_{\mathrm{p}}^{\mathrm{t}}$ are capture rate for electrons and holes, and N_{t} is defect density. The emission rate for electrons and holes ($e_{\mathrm{n}}^{\mathrm{t}}$ and $e_{\mathrm{p}}^{\mathrm{t}}$) are defined as $e_n = \beta_n^{\mathrm{t}} N_{\mathrm{C}} \exp\left(\frac{E_{\mathrm{t}} - E_{\mathrm{C}}}{k_{\mathrm{B}}T}\right)$ and $e_p = \beta_p^{\mathrm{t}} N_{\mathrm{V}} \exp\left(\frac{E_{\mathrm{V}} - E_{\mathrm{t}}}{k_{\mathrm{B}}T}\right)$. Here, N_{V} and N_{C} are the effective density of states for the valance and conduction band, k_{B} the Boltzmann constant, T the temperature, E_{V} and E_{C} the valance and conduction band energy, and E_{t} the trap energy. The occupied defect density (n_{t}) is defined as $n_{\mathrm{t}} = N_{\mathrm{t}} / \left(1 + \exp\left(\frac{E_{\mathrm{V}} - E_{\mathrm{t}}}{k_{\mathrm{B}}T}\right)\right)$.

Note that because of over-all charge conservation we have

$$\frac{\mathrm{d}n}{\mathrm{d}t} + \sum_{j=1}^{3} \frac{\mathrm{d}n_{\mathrm{t}_j}}{\mathrm{d}t} = \frac{\mathrm{d}p}{\mathrm{d}t} \tag{7}$$

An iterative process is used to fit the numerical solution of Eq. 5, 6, and 7 such that the mean squared error (MSE) reduces and no longer changes.

$$\mathrm{MSE} = \frac{\sum\left(\tau_{\mathrm{diff}}^{\mathrm{exp.}} - \tau_{\mathrm{diff}}^{\mathrm{sim.}}\right)^2}{\sum\left(\tau_{\mathrm{diff}}^{\mathrm{exp.}}\right)^2} \tag{8}$$

Since for each defect four fitting parameters (N_{t}, E_{t}, β_{n}, β_{p}) can be adjusted, that results in 13 degrees of freedom when also considering k_{rad}. This allows the iterative process to find unphysical fitting parameters to obtain a low MSE, for example $E_{\mathrm{t}} > E_{\mathrm{g}}$. Hence, a constraint on E_{t} was set so it cannot be larger than E_{g} − 0.01 eV.

Supplementary references

(1) Mooney, J.; Kambhampati, P. Get the Basics Right: Jacobian Conversion of Wavelength and Energy Scales for Quantitative Analysis of Emission Spectra. *J. Phys. Chem. Lett.* **2013**, *4* (19), 3316–3318. https://doi.org/10.1021/jz401508t.

(2) Siebentritt, S.; Weiss, T. P.; Sood, M.; Wolter, M. H.; Lomuscio, A.; Ramirez, O. How Photoluminescence Can Predict the Efficiency of Solar Cells. *J. Phys. Mater.* **2021**, *4* (4), 042010. https://doi.org/10.1088/2515-7639/ac266e.

(3) Luo, D.; Su, R.; Zhang, W.; Gong, Q.; Zhu, R. Minimizing Non-Radiative Recombination Losses in Perovskite Solar Cells. *Nat. Rev. Mater.* **2020**, *5* (1), 44–60. https://doi.org/10.1038/s41578-019-0151-y.

(4) Yuan, Y.; Yan, G.; Dreessen, C.; Rudolph, T.; Hülsbeck, M.; Klingebiel, B.; Ye, J.; Rau, U.; Kirchartz, T. Shallow Defects and Variable Photoluminescence Decay Times up to 280 Ms in Triple-Cation Perovskites. *Nat. Mater.* **2024**, *23* (3), 391–397. https://doi.org/10.1038/s41563-023-01771-2.
